# Supplementary material for: Novel Galactopyranoside Esters: Synthesis, Mechanism, In Vitro Antimicrobial Evaluation and Molecular Docking Studies
Source: Molecules. 2022 Jun 27;27(13):4125. doi: 10.3390/molecules27134125 (PMC9268324; doi:10.3390/molecules27134125)
Supplement: Supplementary file 1 [file molecules-27-04125-s001.zip › molecules-1735770-supplementary.pdf]

Supplementary Information (SI)

Novel Galactopyranoside Esters: Synthesis, Mechanism, *In Vitro* Antimicrobial Evaluation, and Molecular Docking Studies

Table of contents

- FT-IR spectra
- ^{1}H NMR spectra (with expansion)
- ^{13}C NMR spectra (with expansion)
- 2D COSY spectra
- 2D HSQC spectra
- 2D HMBC spectra
- Antibacterial activity test picture
- Antifungal activity test picture
- MM-GBSA Binding affinity calculation (Table S1 and S2)

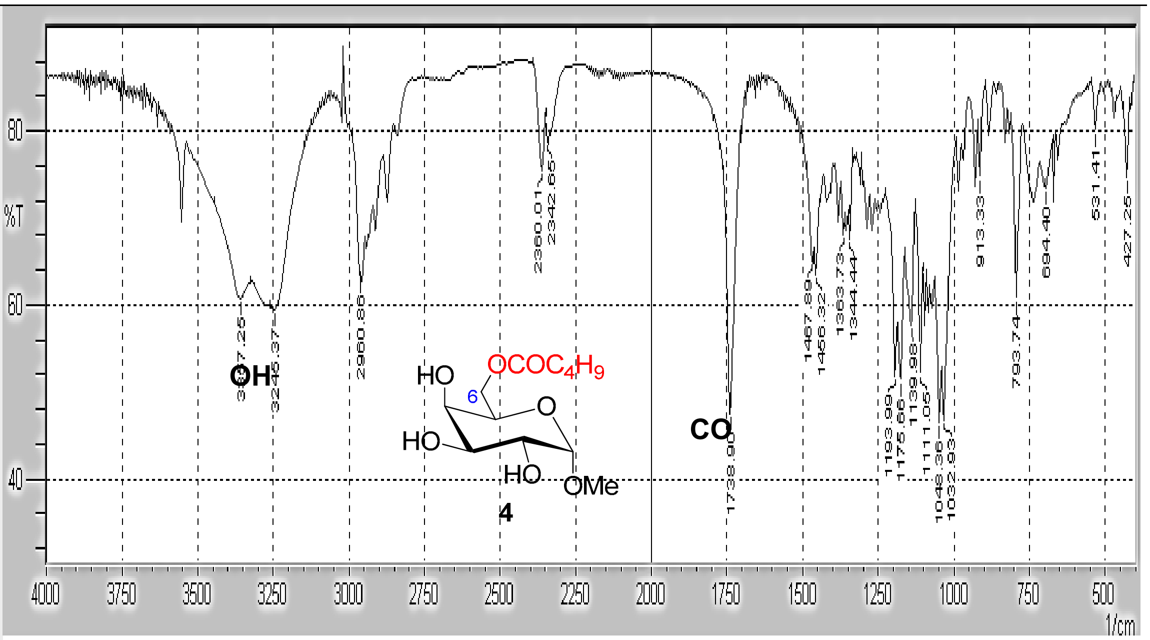

Figure S1. FT-IR (KBr) spectrum of compound **4**.

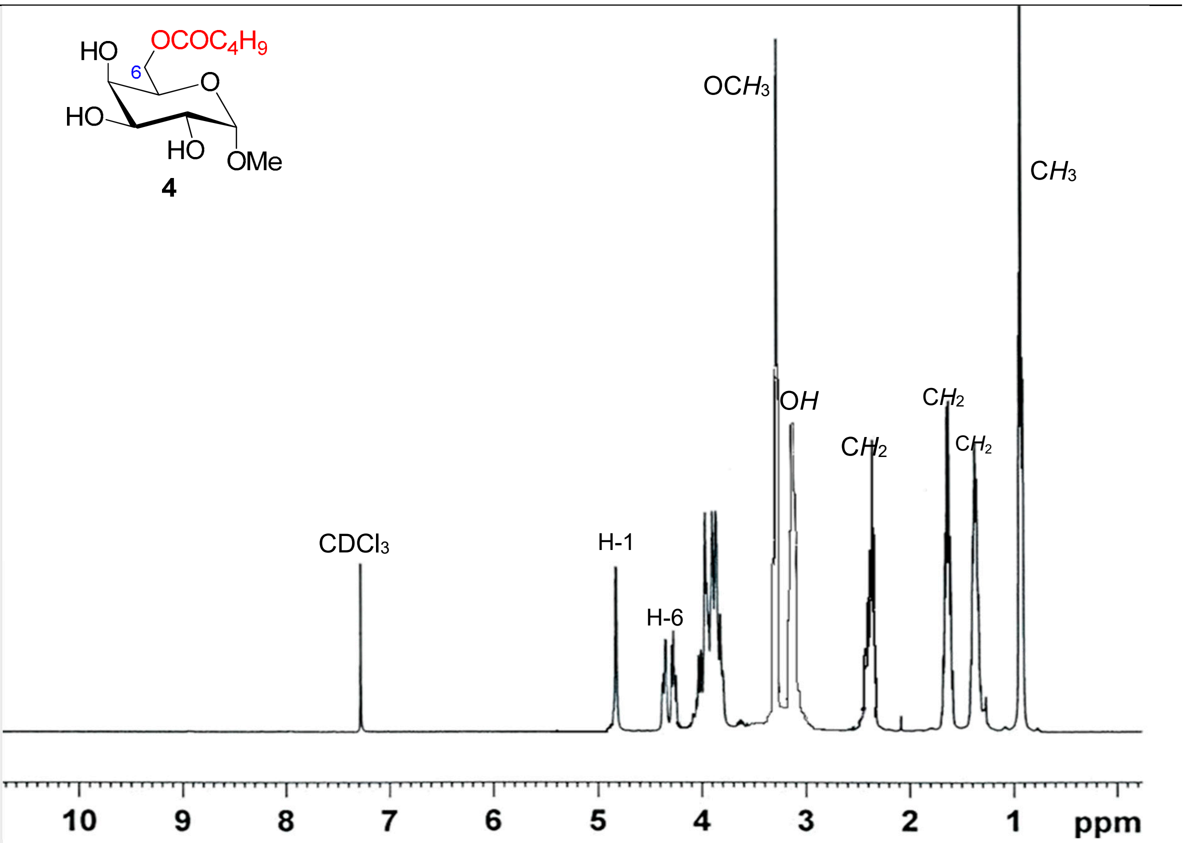

Figure S2. ¹H NMR (400 MHz, $CDCl_3$) spectrum of compound **4.**

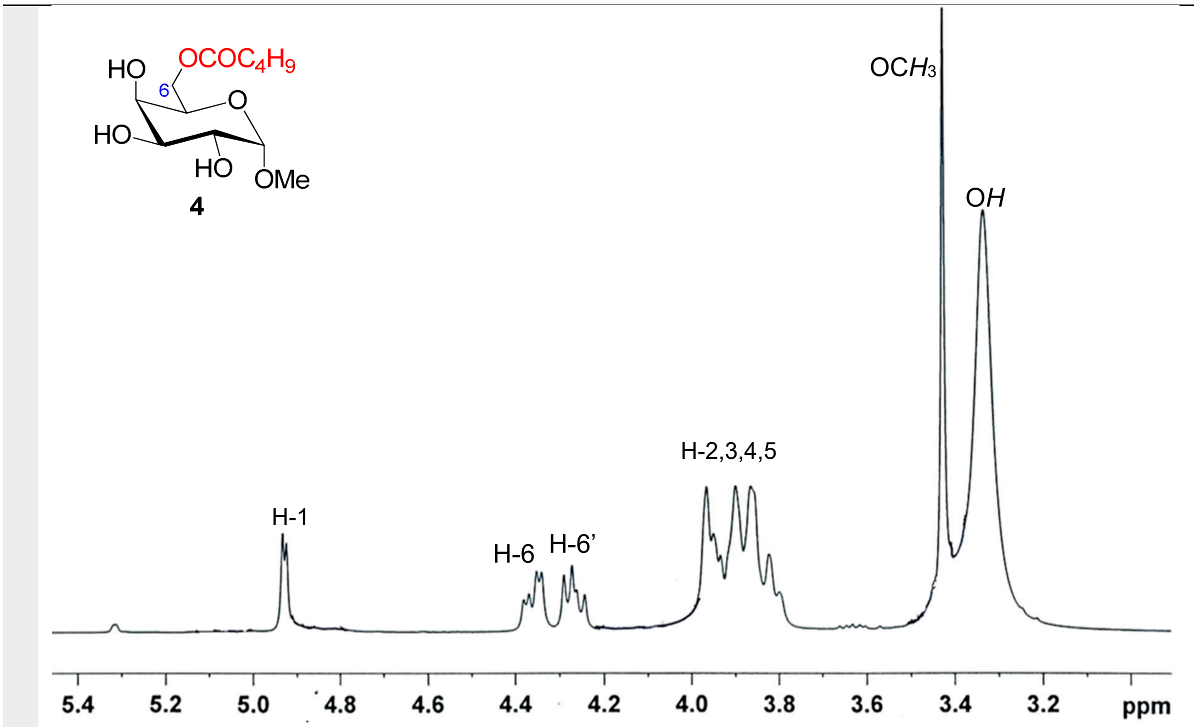

Figure S3. ^{1}H NMR (400 MHz, $CDCl_3$) spectrum of compound **4** (expansion).

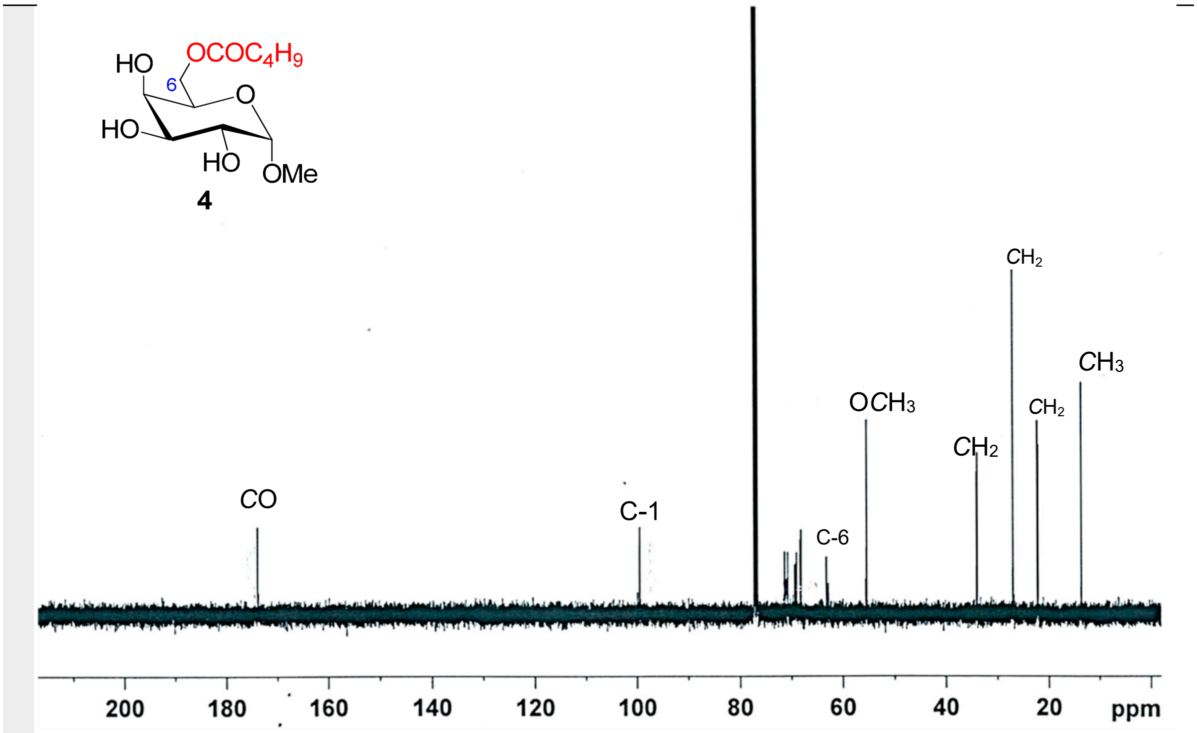

Figure S4. ^{13}C NMR (100 MHz, $CDCl_3$) spectrum of compound **4**.

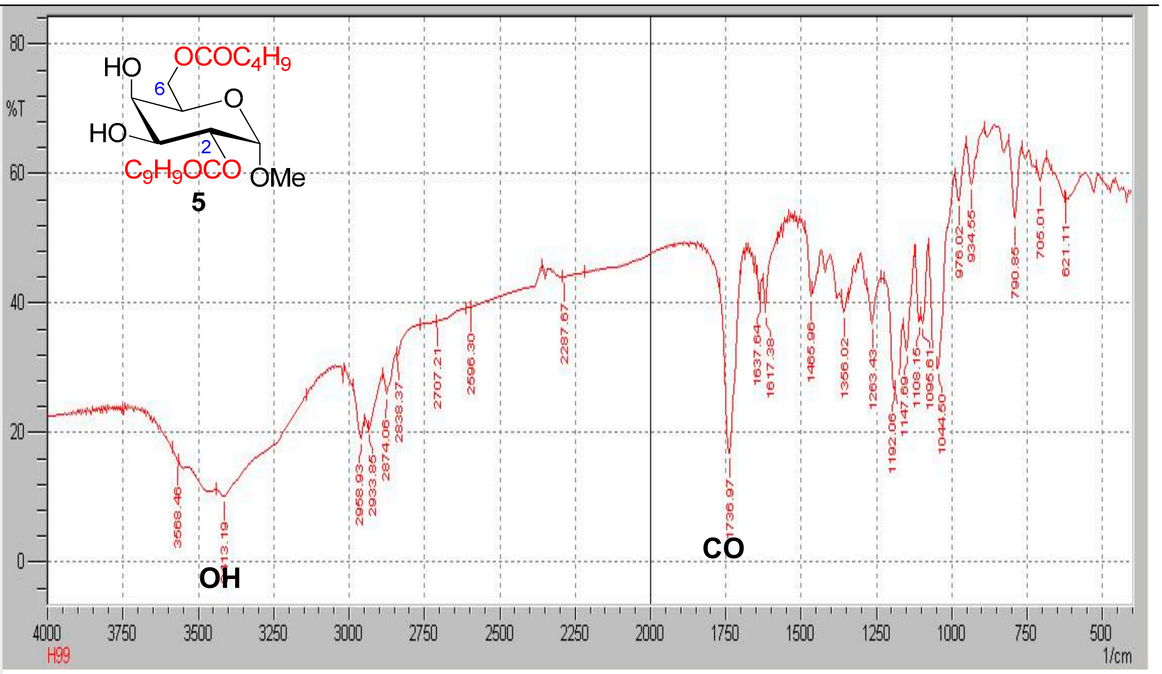

Figure S5. FT-IR (KBr) spectrum of compound **5**.

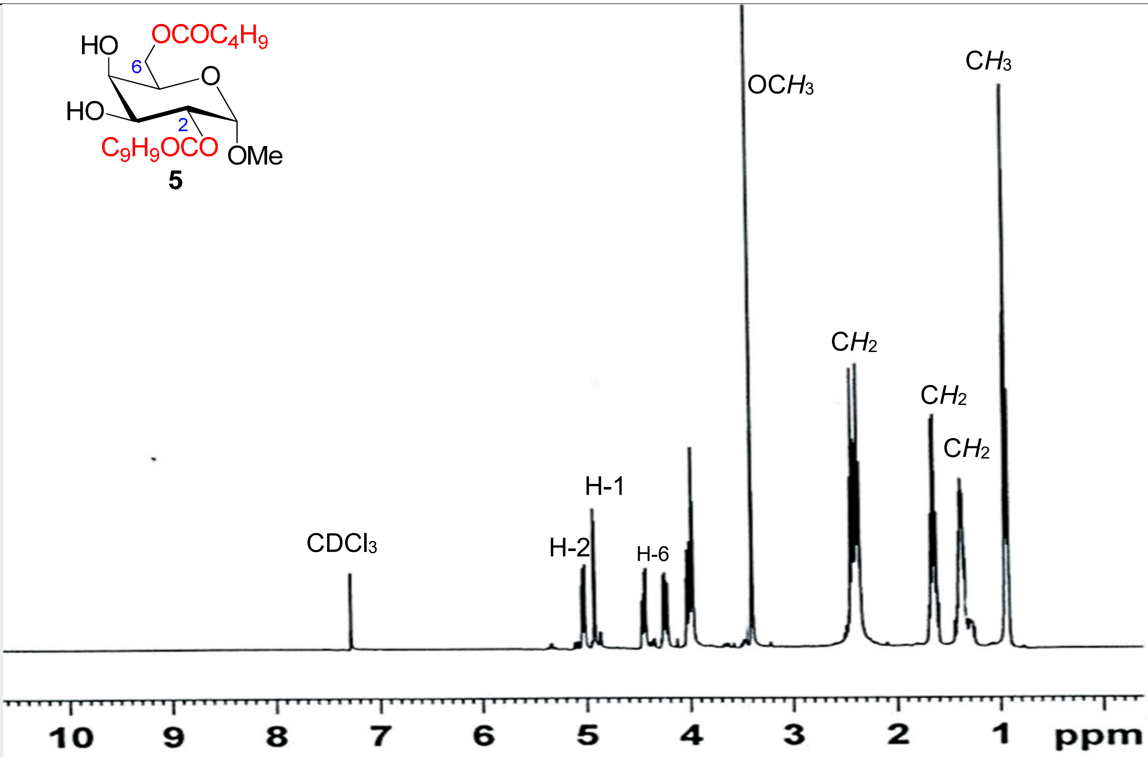

Figure S6. ^{1}H NMR (400 MHz, $CDCl_3$) spectrum of compound **5**.

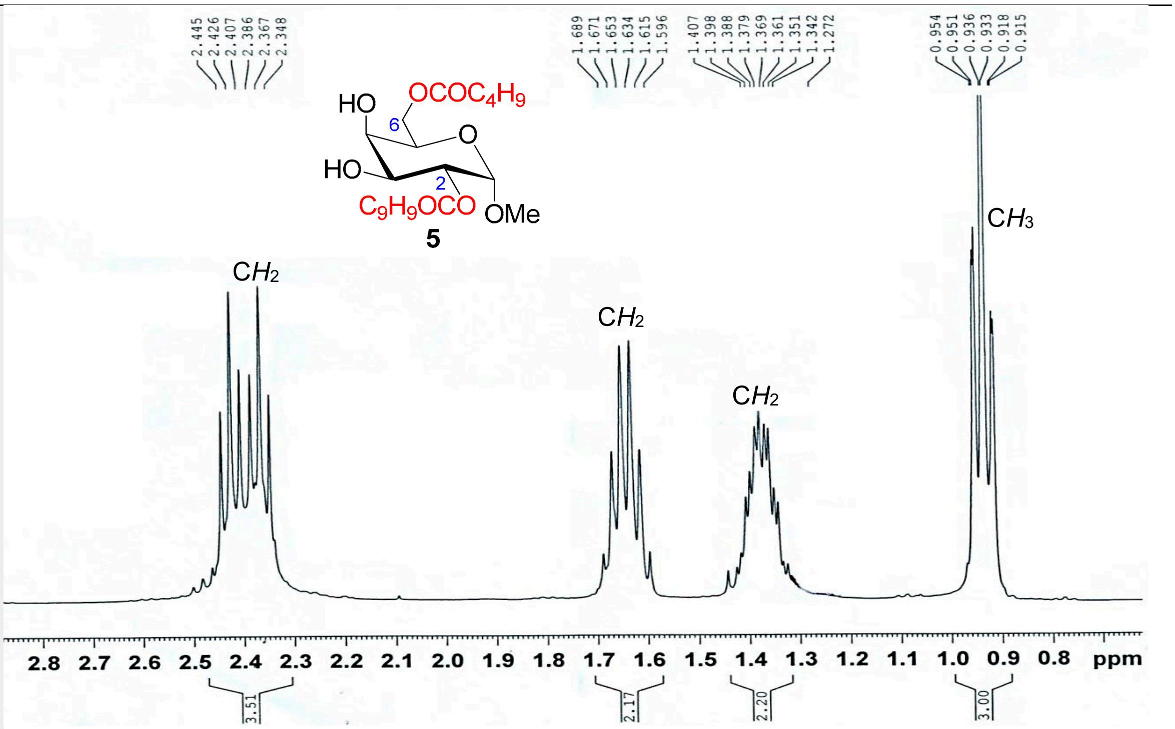

Figure S7. ^{1}H NMR (400 MHz, $CDCl_3$) spectrum of compound **5** (expansion).

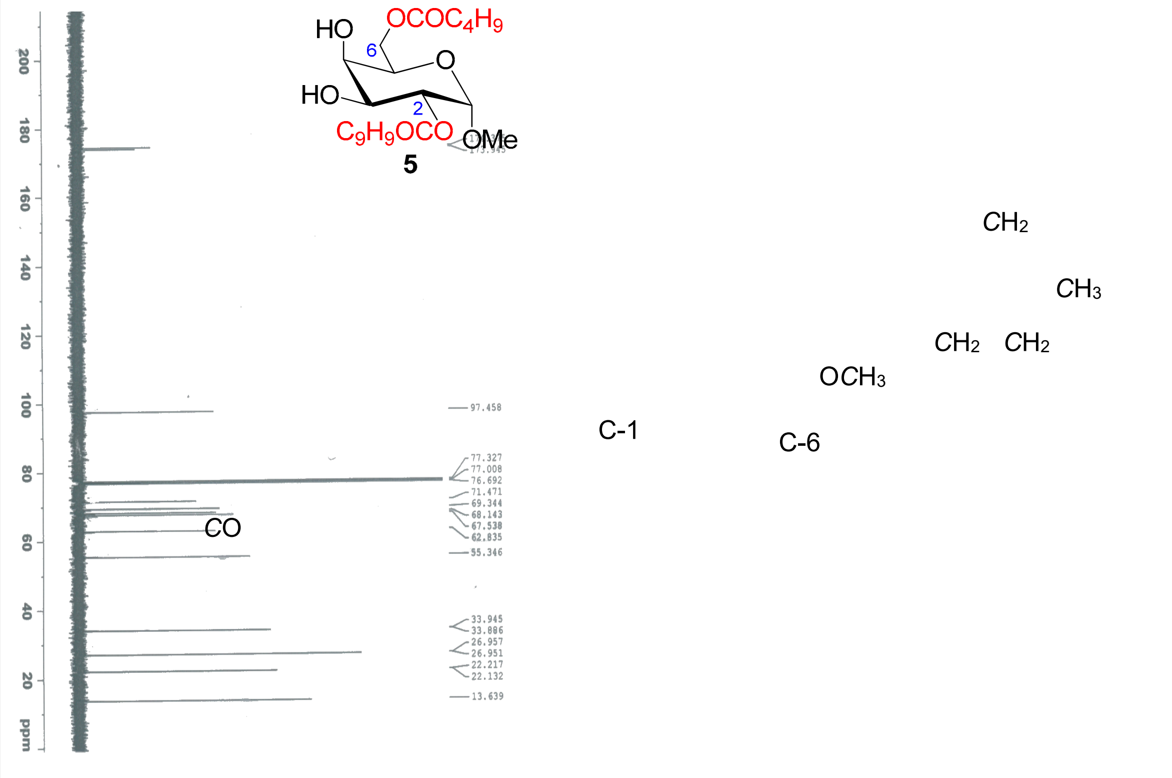

Figure S8. ^{13}C NMR (100 MHz, $CDCl_3$) spectrum of compound **5**.

The assignments of the signals of this compound **5** were established by analyzing its COSY (Figure **S9-S10**) and HMBC (Figure **S11-S12**) experiments.

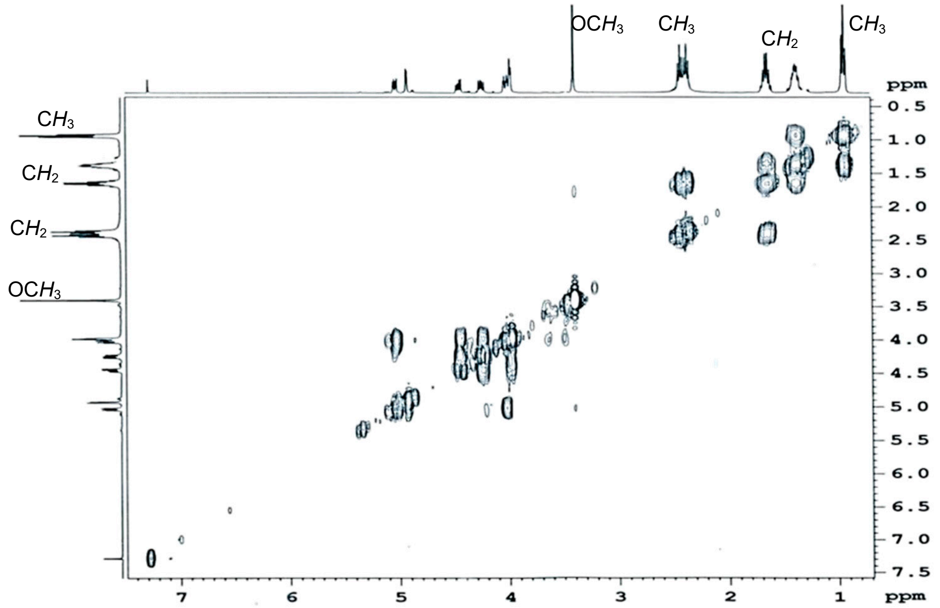

Figure S9. 2D COSY spectrum of compound **5**.

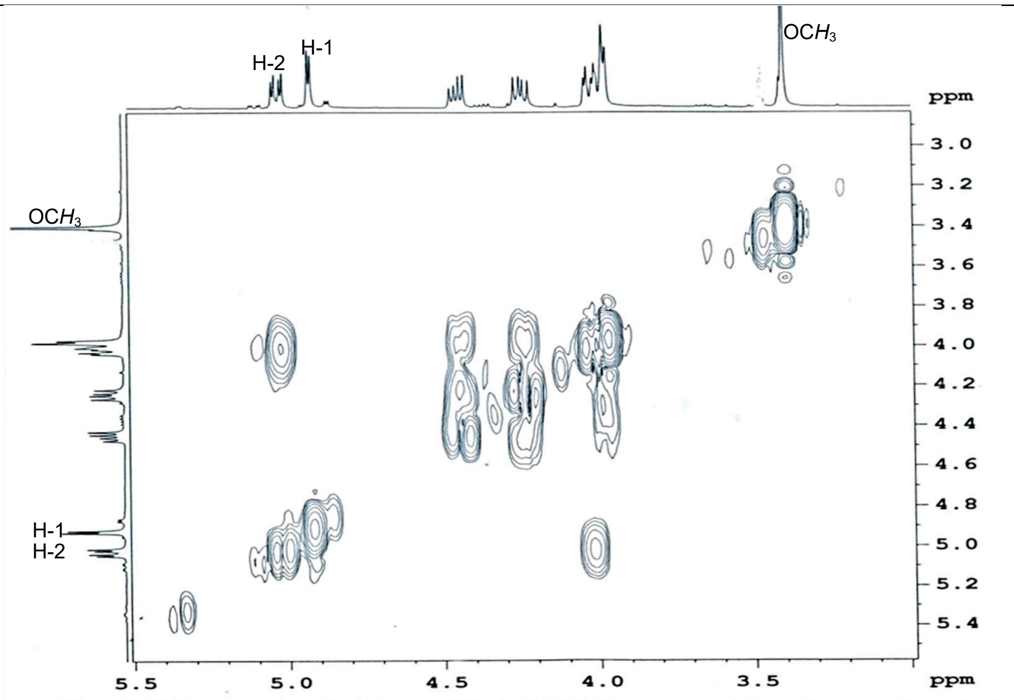

Figure S10. COSY spectrum of compound **5** (expansion).

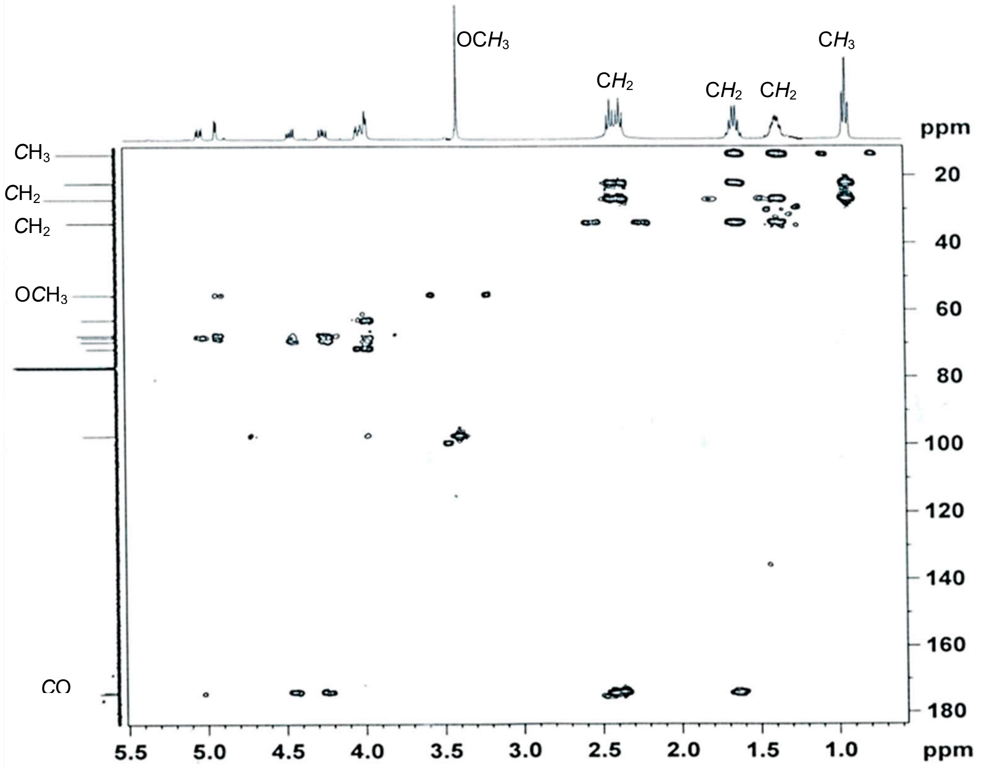

Figure S11. HMBC spectrum of compound **5**.

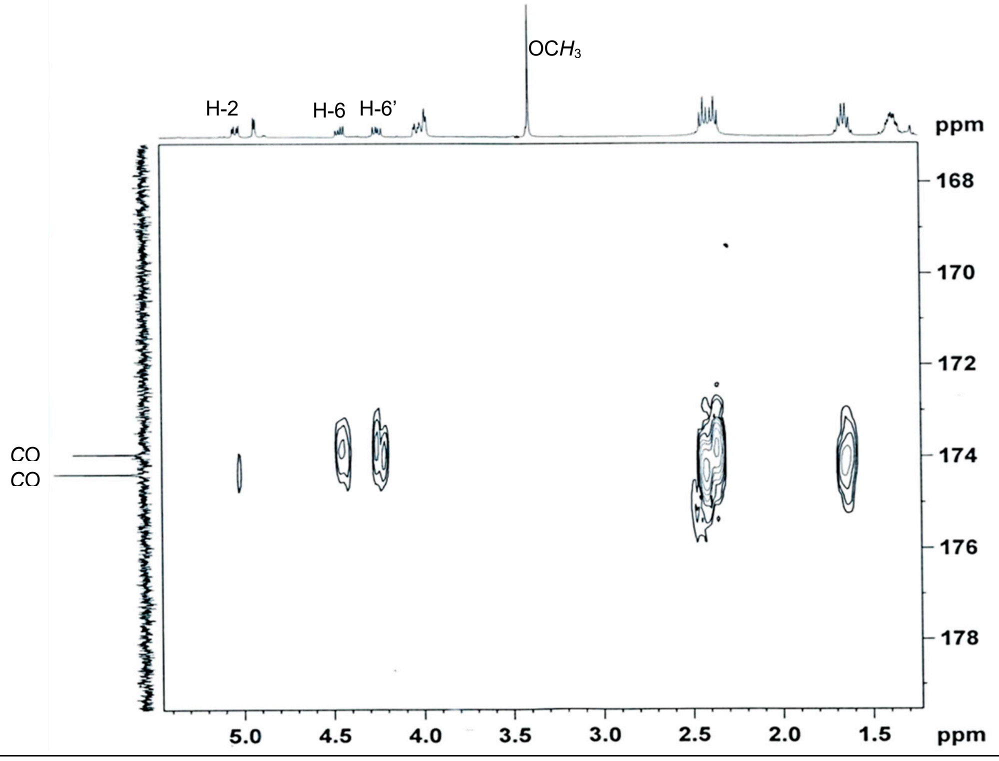

Figure S12. HMBC spectrum of compound **5** (expansion).

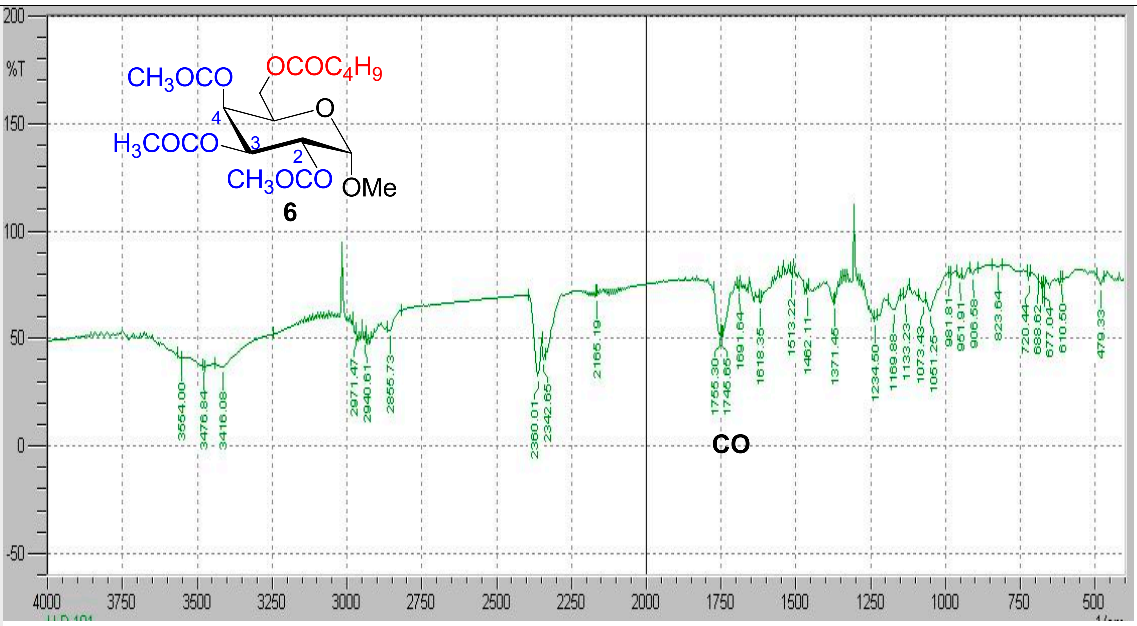

Figure S13. FT-IR (KBr) spectrum of compound **6**.

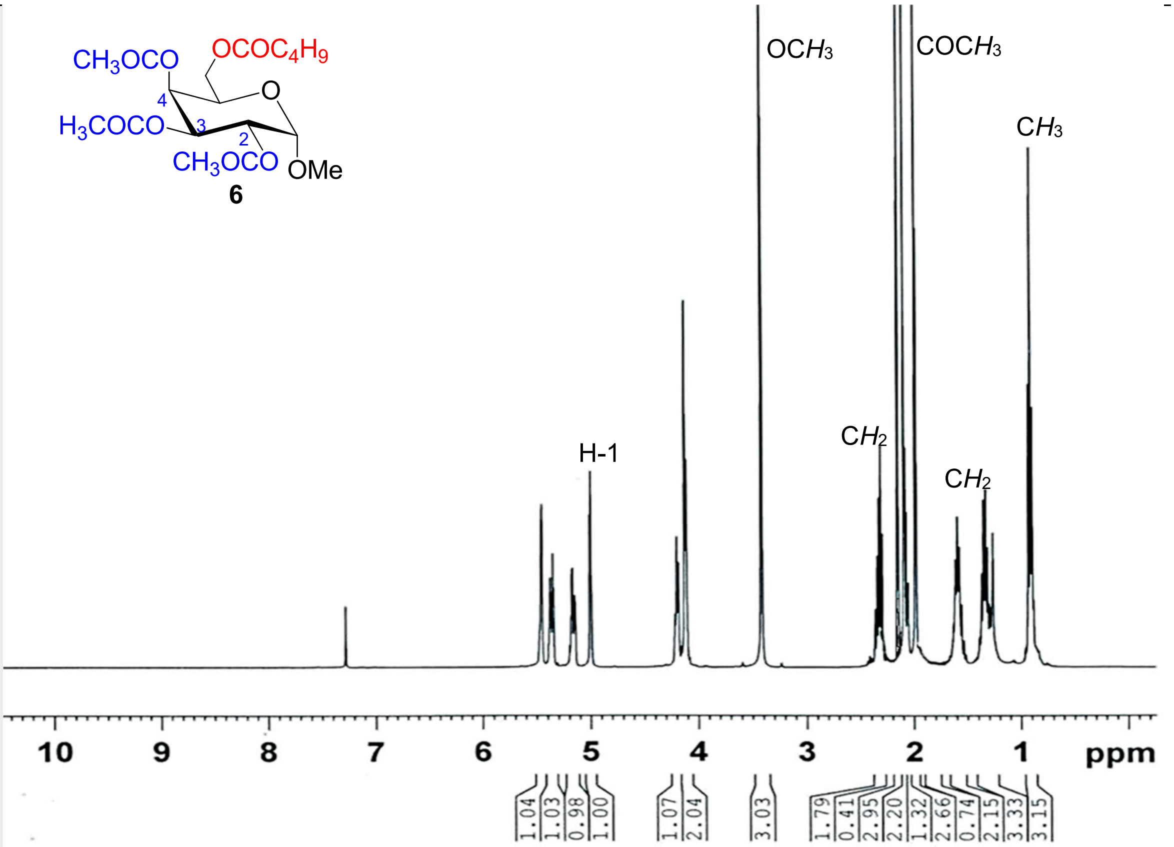

Figure S14. ^{1}H NMR (400 MHz, $CDCl_3$) spectrum of compound **6.**

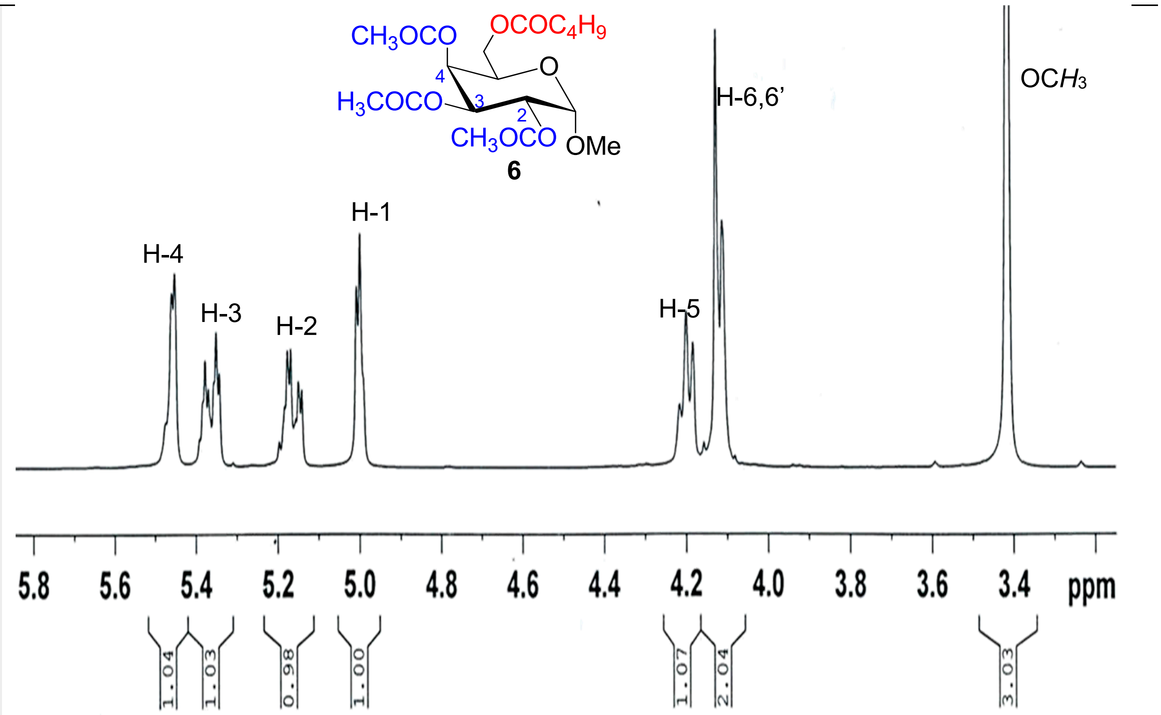

Figure S15. ^{1}H NMR (400 MHz, $CDCl_3$) spectrum of compound **6** (expansion).

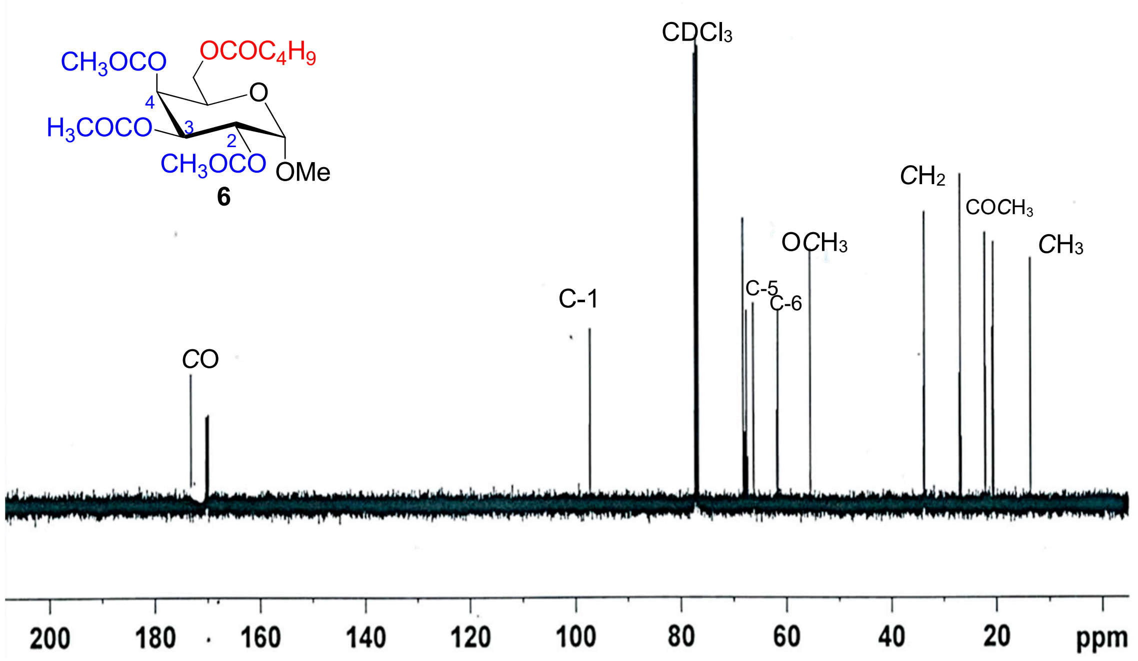

Figure S16. ^{13}C NMR (100 MHz, $CDCl_3$) spectrum of compound **6**.

The assignments of the signals of this compound **6** were established by analyzing its COSY (Figure 17), HSQC (Figure 18) and HMBC (Figure 19-20) experiments.

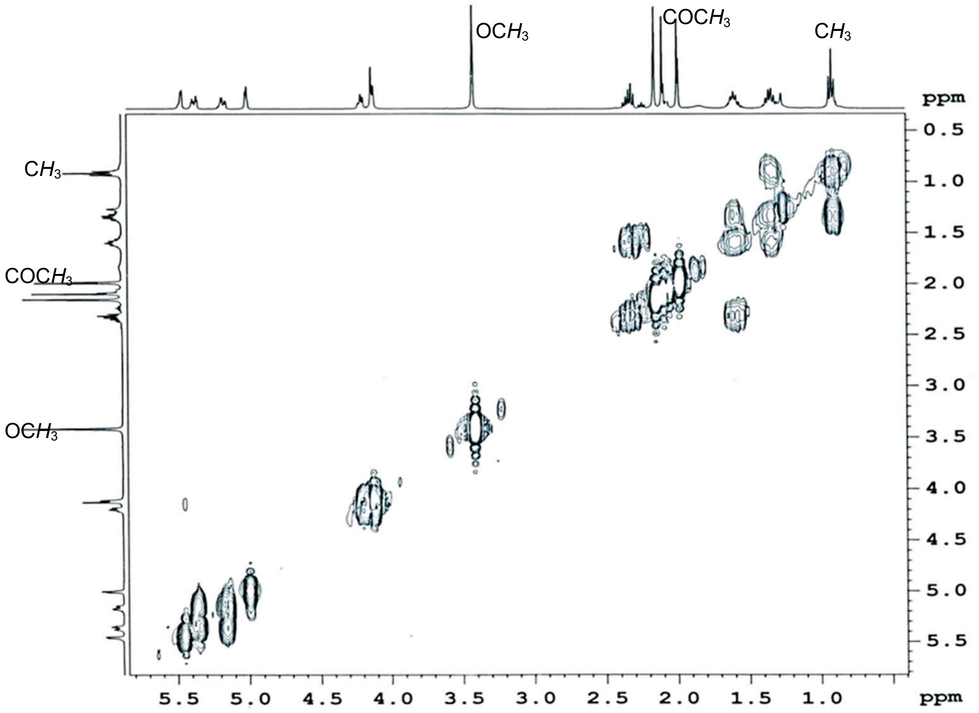

Figure S17. 2D COSY spectrum of compound **6**.

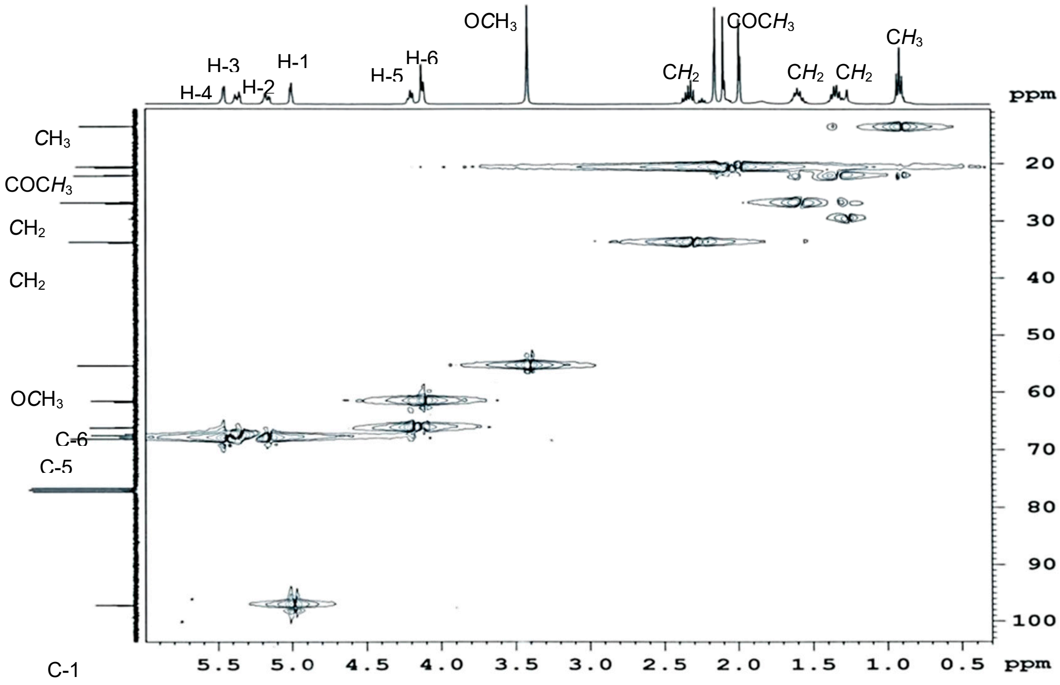

Figure S18 2D HSQC spectrum of compound **6**.

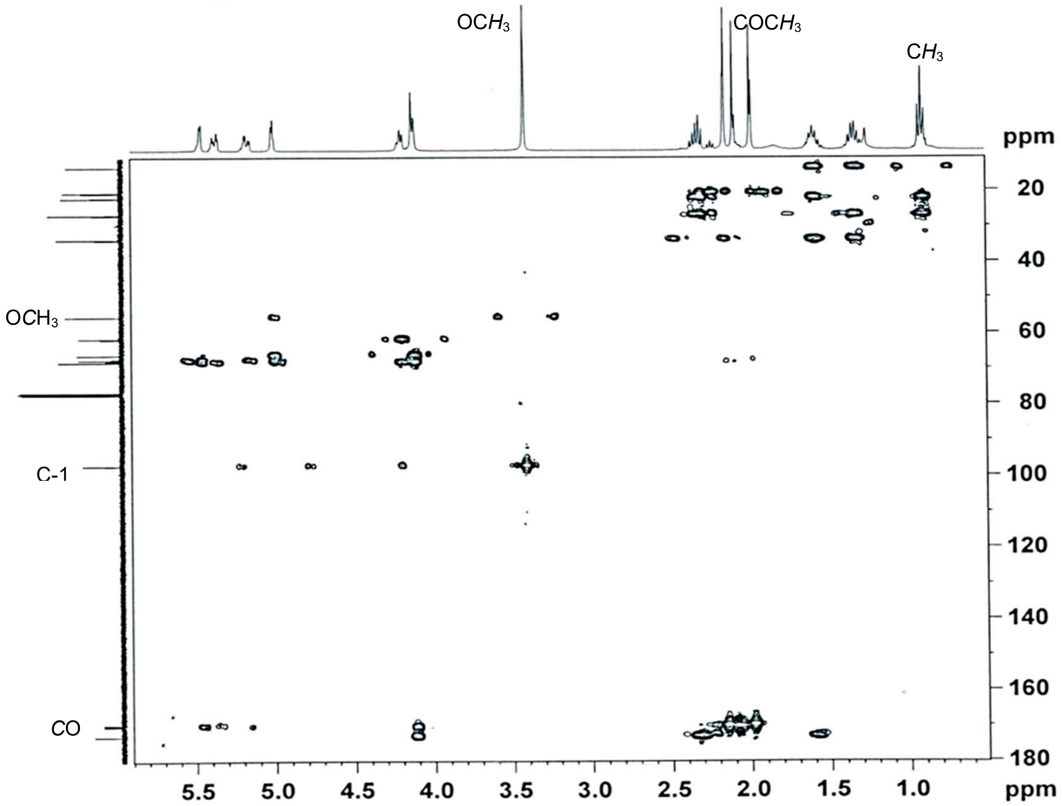

Figure S19. 2D HMBC spectrum of compound **6**.

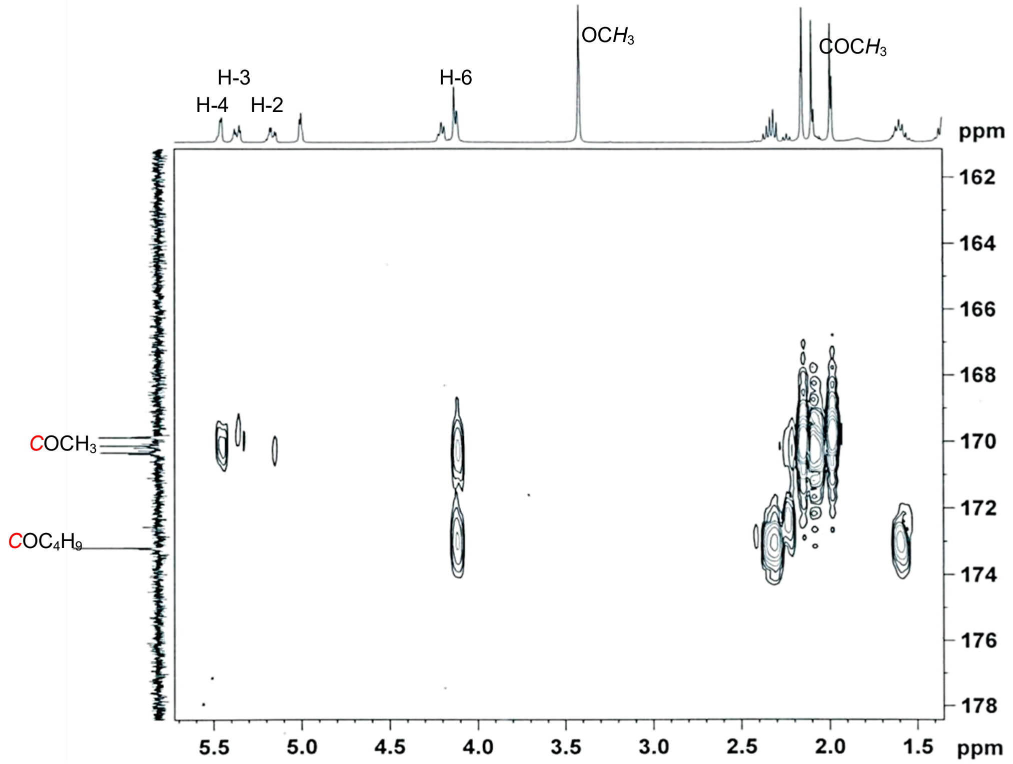

Figure S20. 2D HMBC spectrum of compound **6** (expansion).

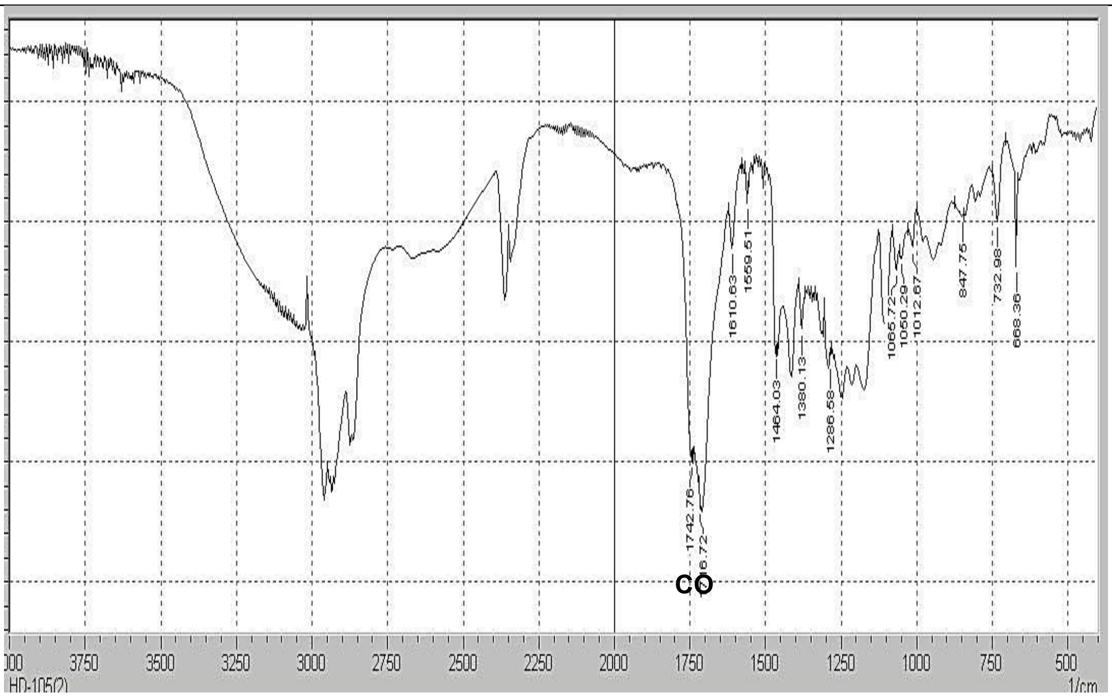

Figure S21. FT-IR (KBr) spectrum of compound **7**.

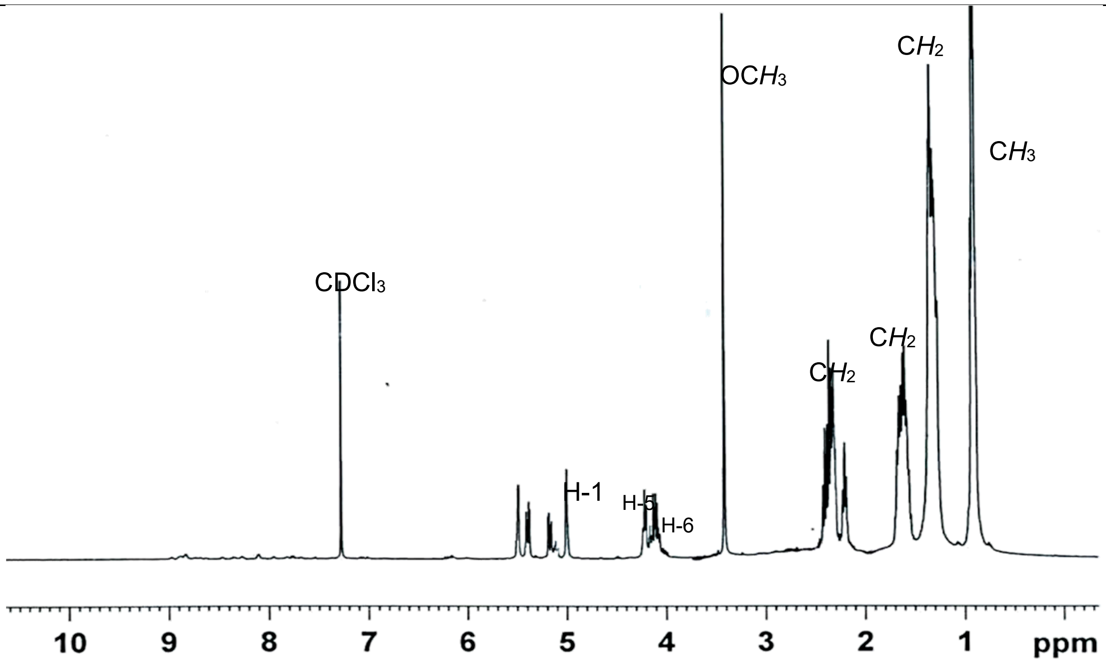

Figure S22. ^{1}H NMR (400 MHz, $CDCl_3$) spectrum of compound **7.**

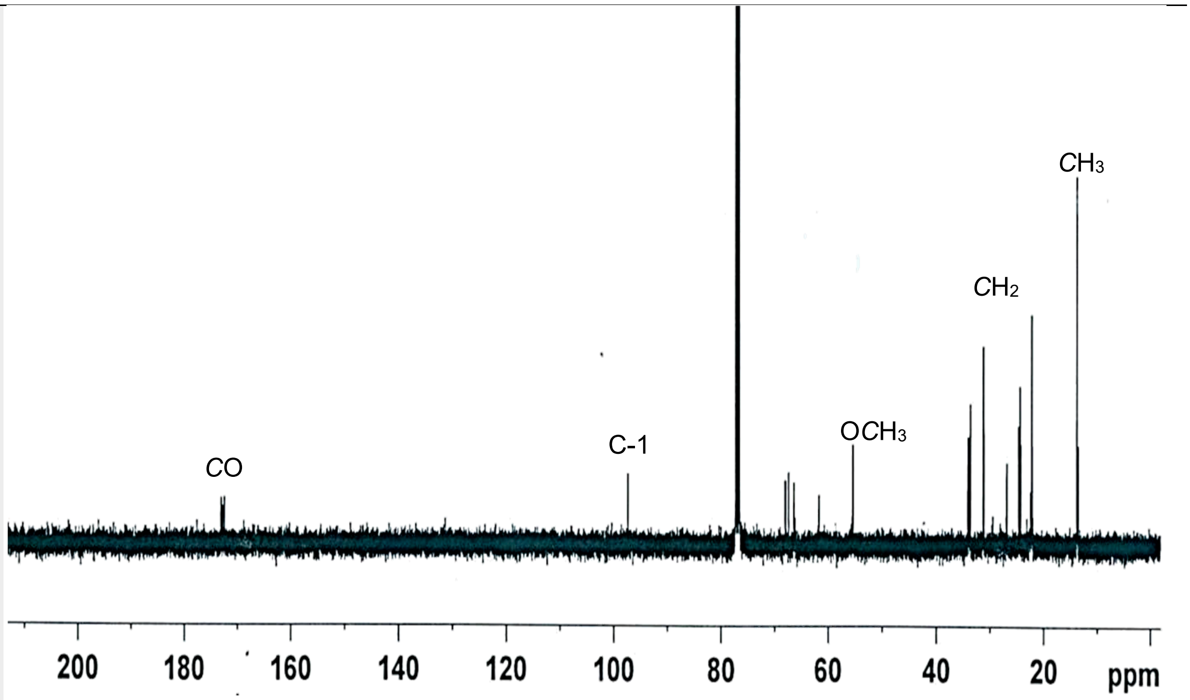

Figure S23. ^{13}C NMR (100 MHz, $CDCl_3$) spectrum of compound **7**.

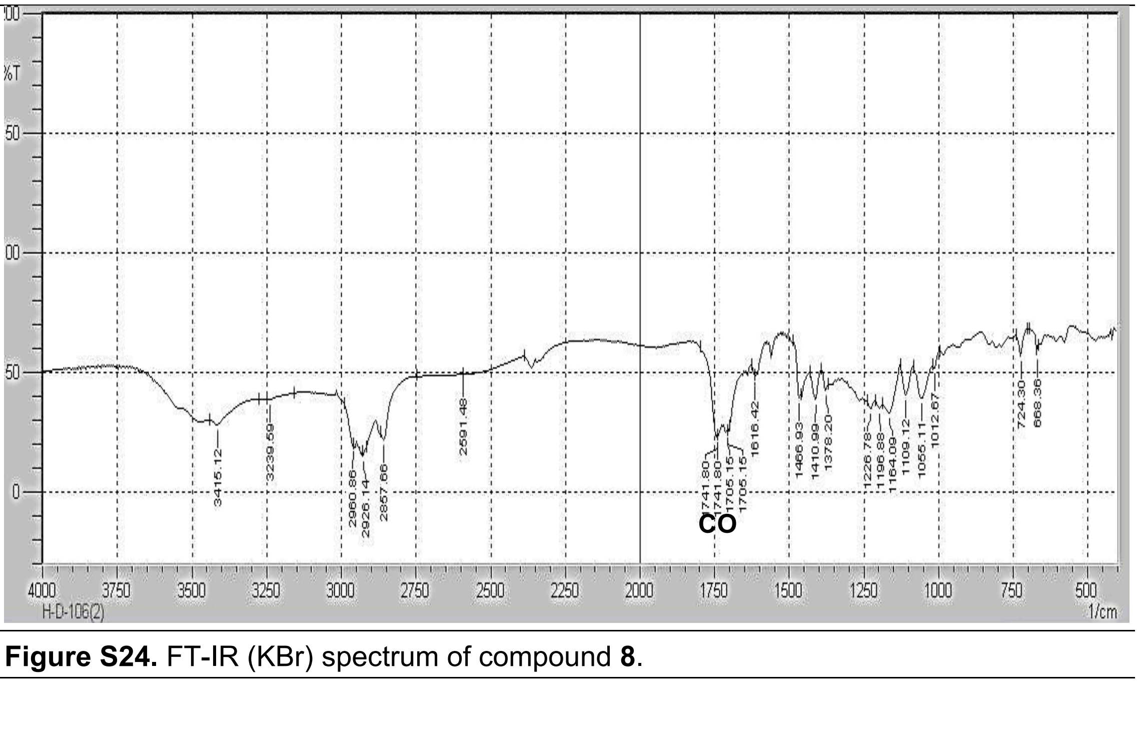

Figure S24. FT-IR (KBr) spectrum of compound **8**.

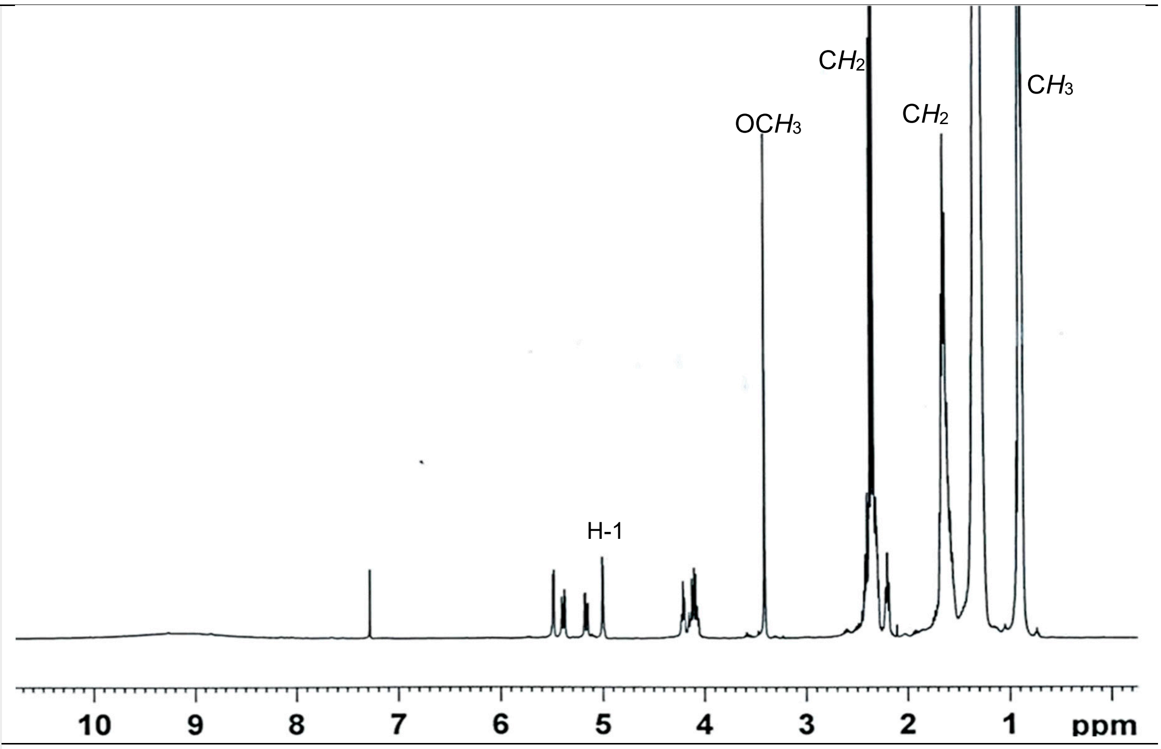

Figure S25. ^{1}H NMR (400 MHz, $CDCl_3$) spectrum of compound **8.**

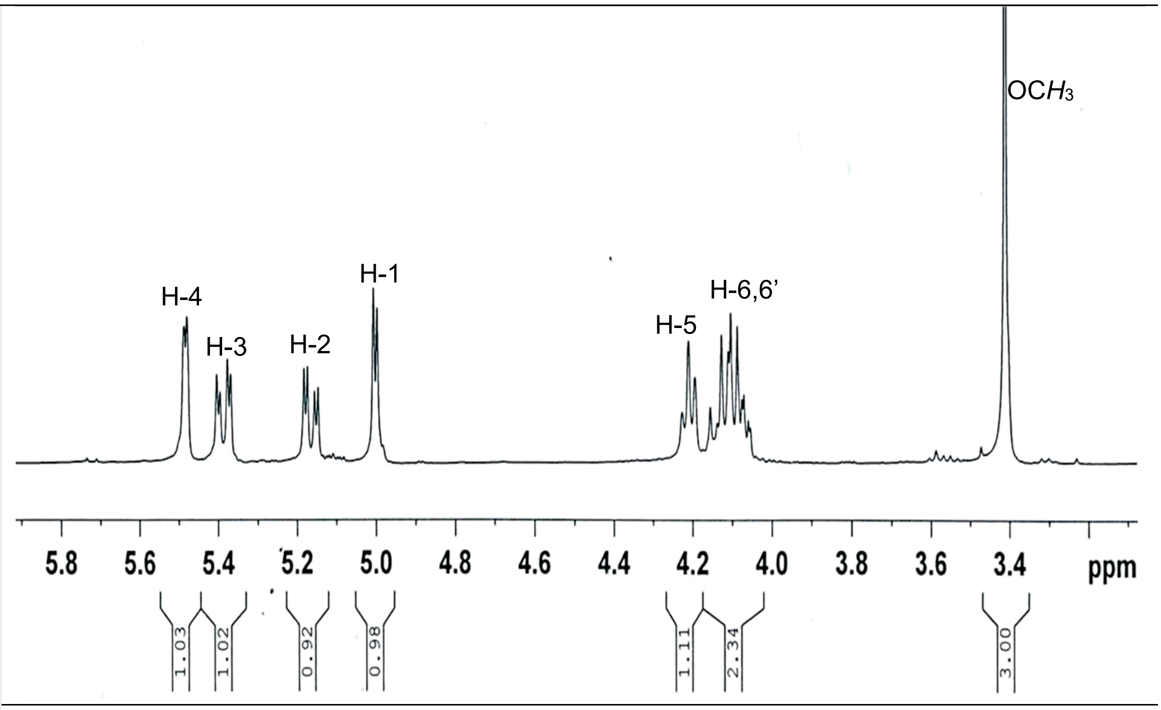

Figure S26. ^{1}H NMR (400 MHz, $CDCl_3$) spectrum of compound **8** (expansion).

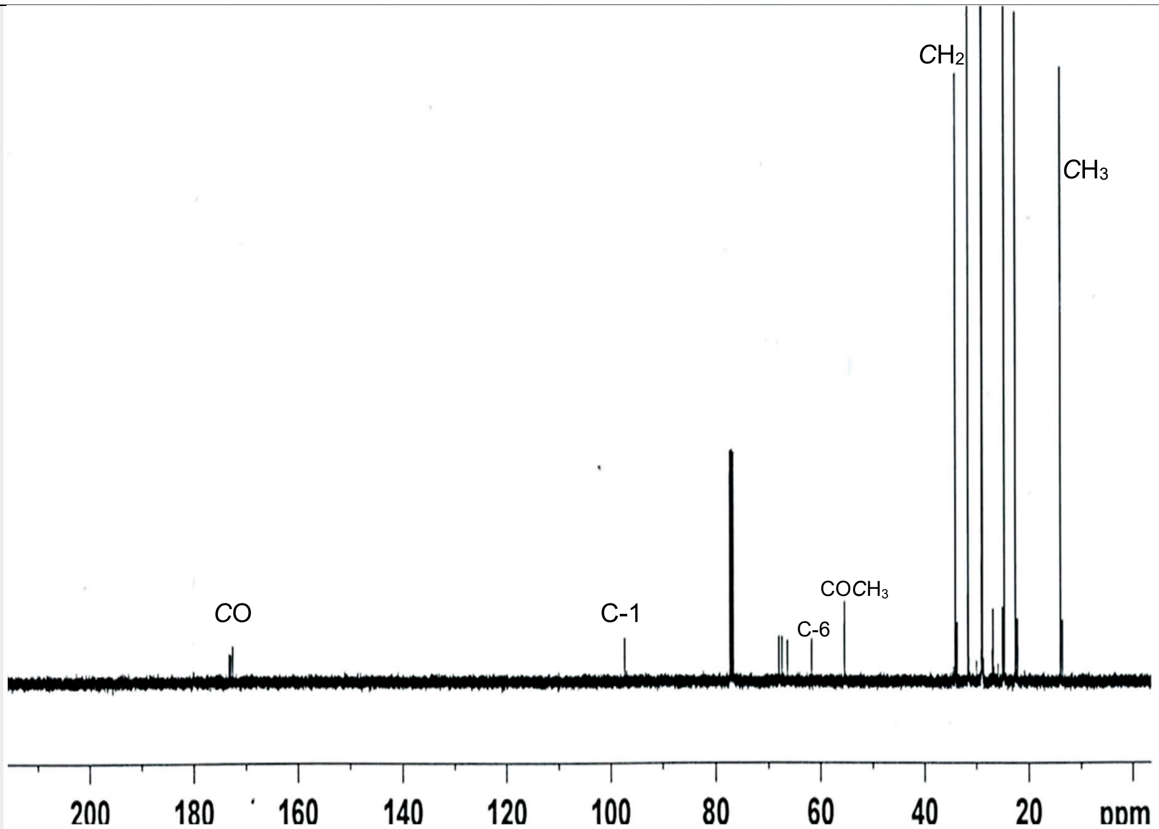

Figure S27. ^{13}C NMR (100 MHz, $CDCl_3$) spectrum of compound **8**.

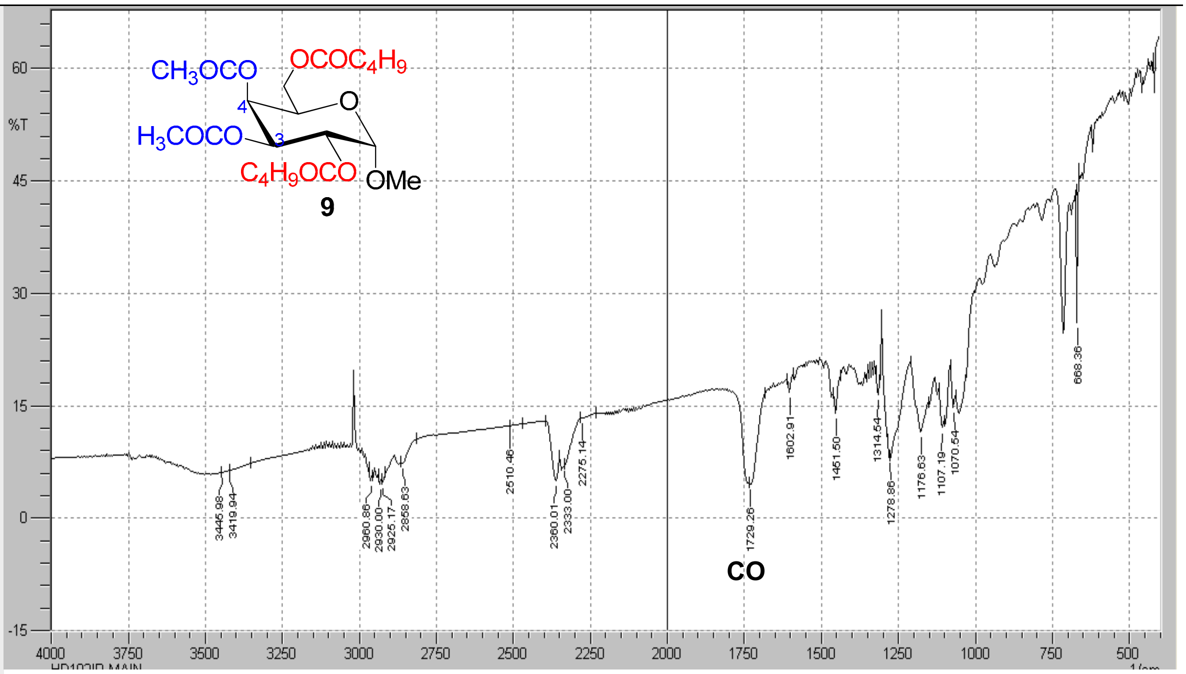

Figure S28. FT-IR (KBr) spectrum of compound **9**.

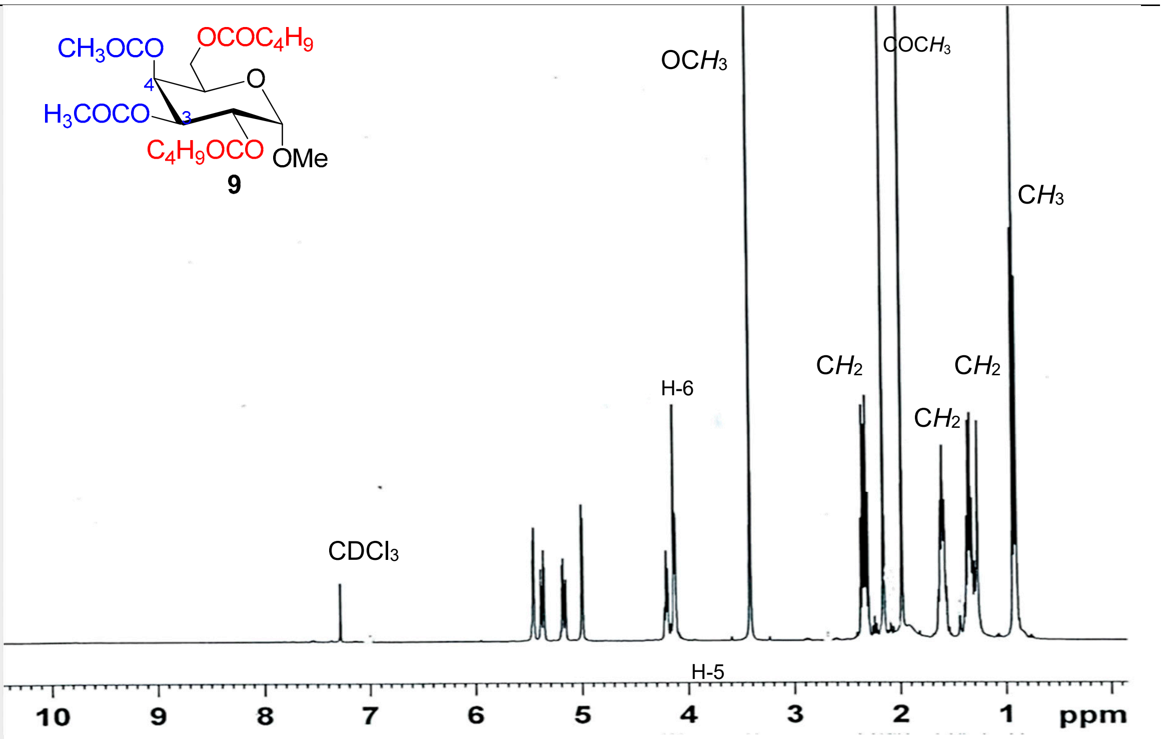

Figure S29. ^{1}H NMR (400 MHz, $CDCl_3$) spectrum of compound **9.**

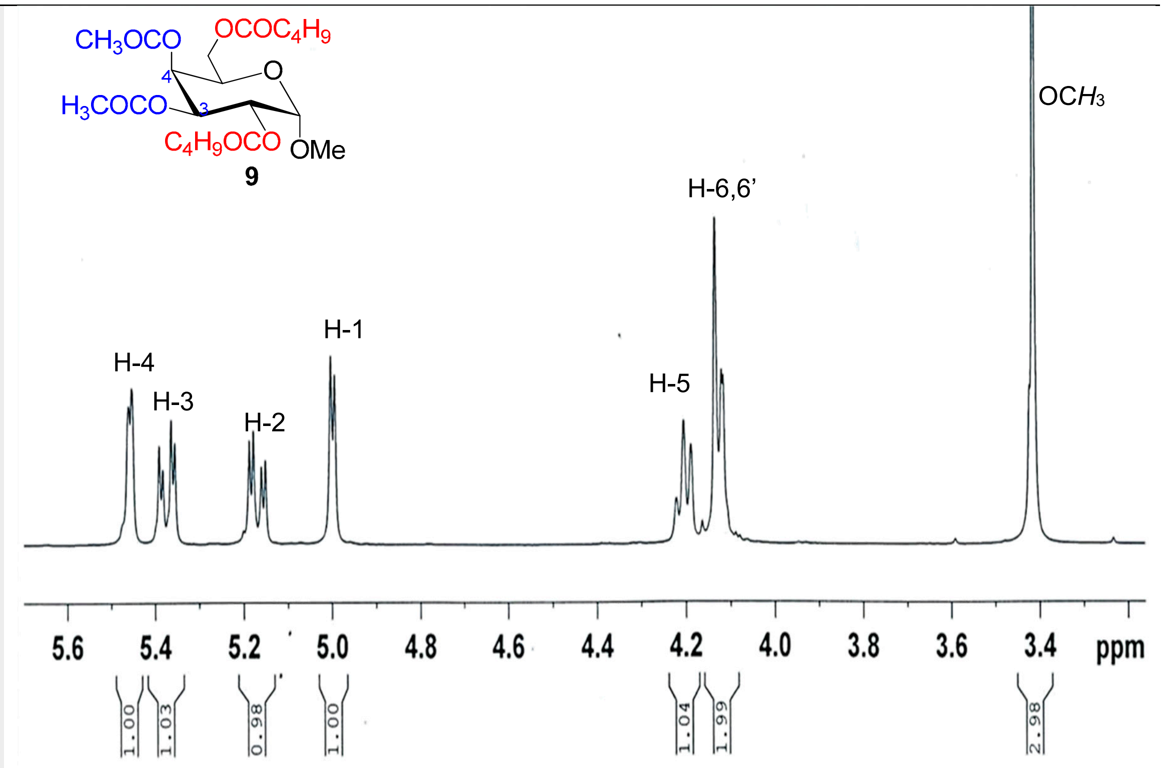

Figure S30. ^{1}H NMR (400 MHz, $CDCl_3$) spectrum of compound **9** (expansion).

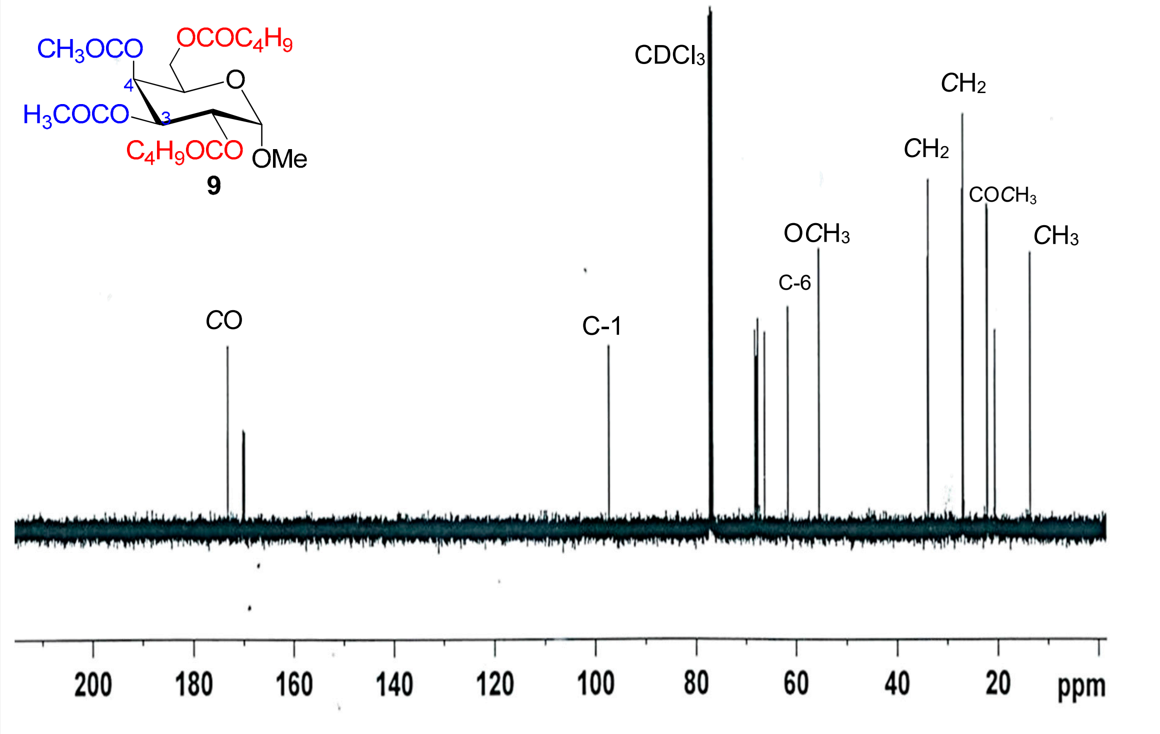

Figure S31. ^{13}C NMR (100 MHz, $CDCl_3$) spectrum of compound **9**.

The assignments of the signals of this compound **9** were established by analyzing its COSY (Figure 32-33), HSQC (Figure 34) and HMBC (Figure 35) experiments.

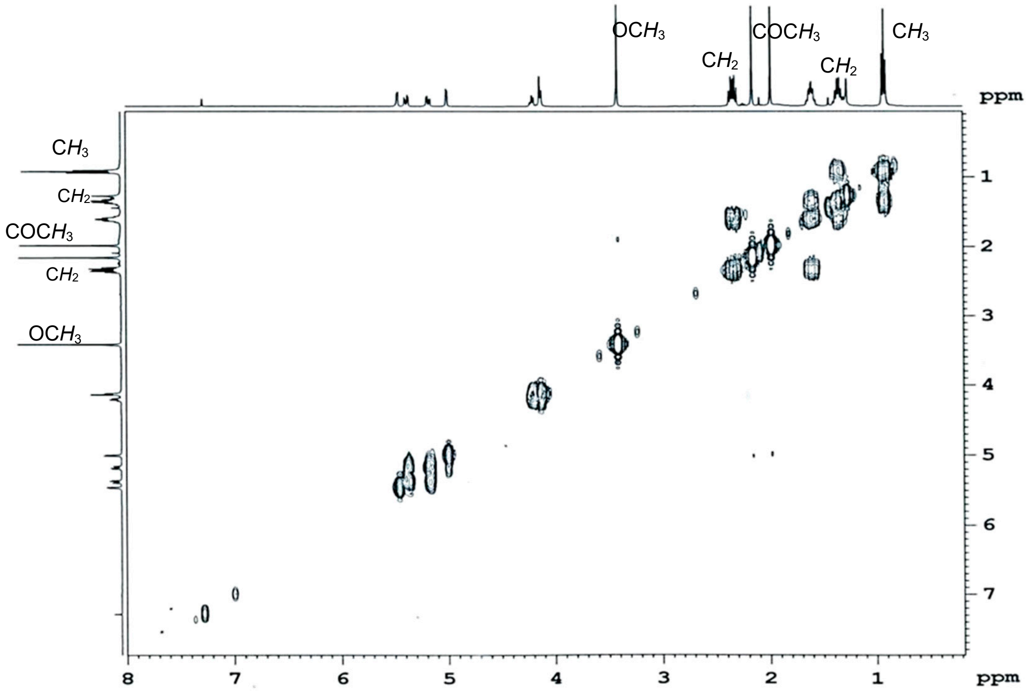

Figure S32. 2D COSY spectrum of compound **9**.

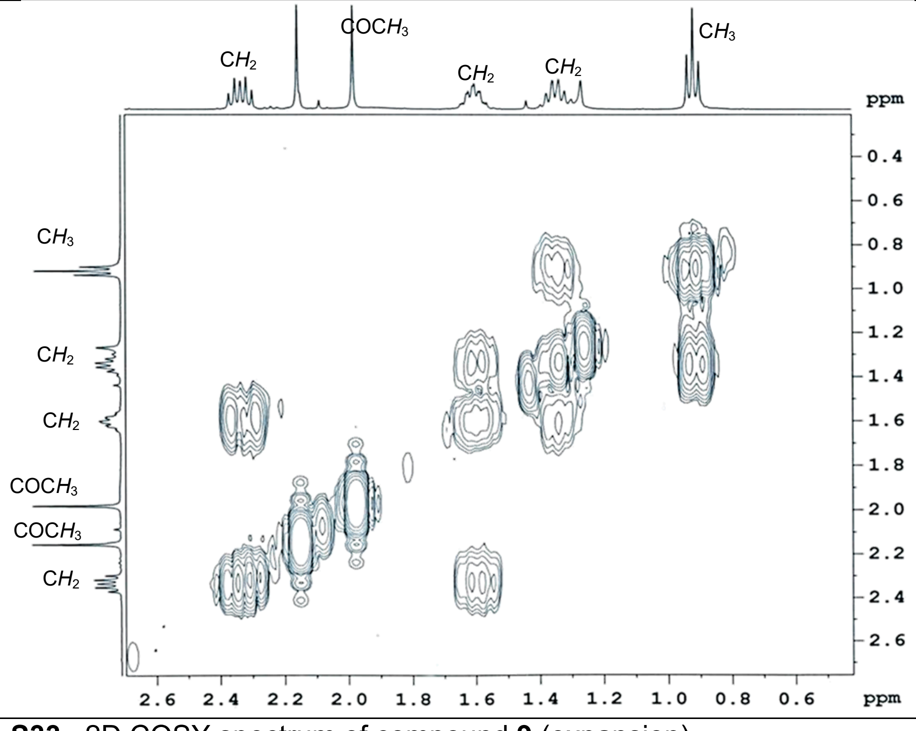

Figure S33. 2D COSY spectrum of compound **9** (expansion).

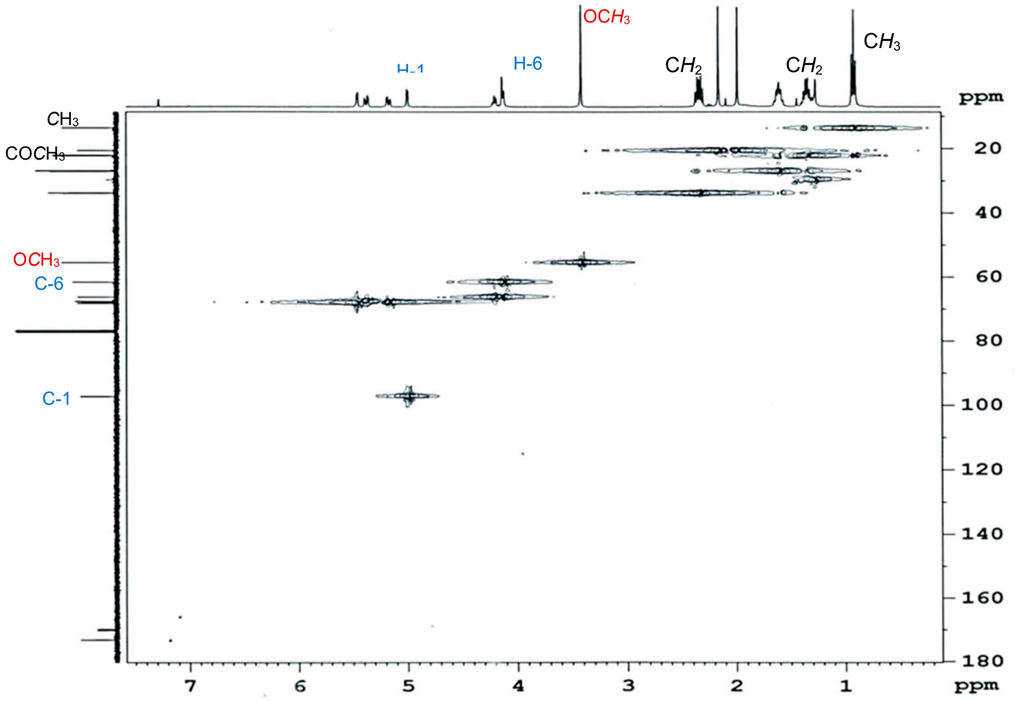

Figure S34. 2D HSQC spectrum of compound **9**.

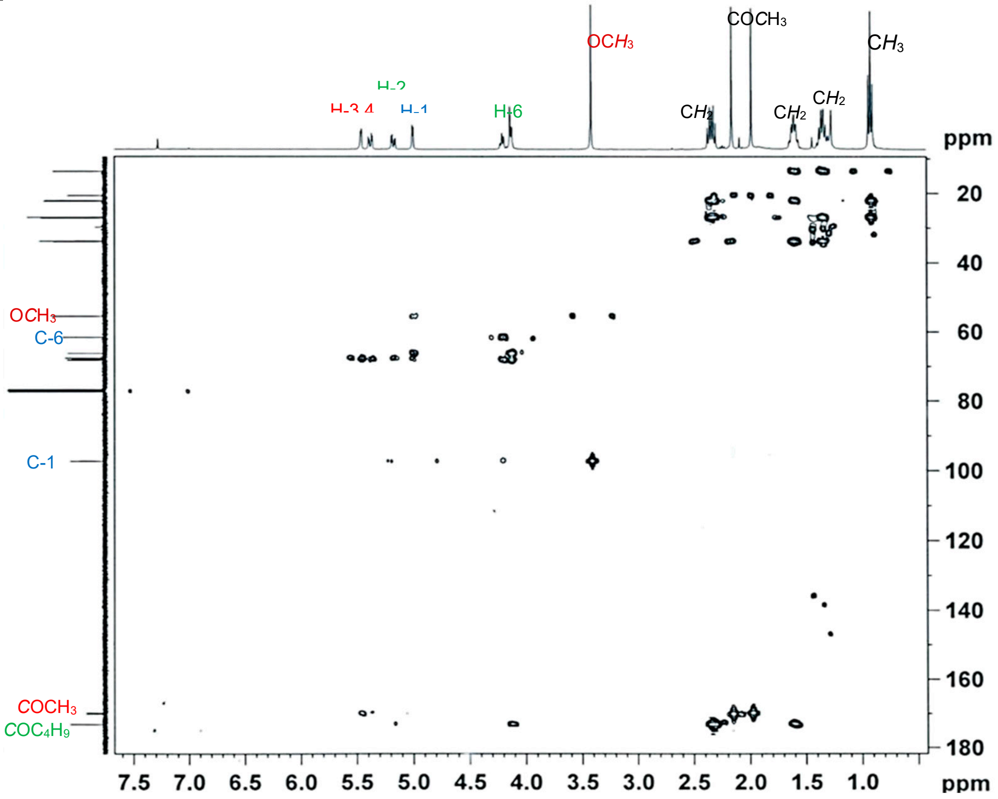

Figure S35. HMBC spectrum of compound **9**.

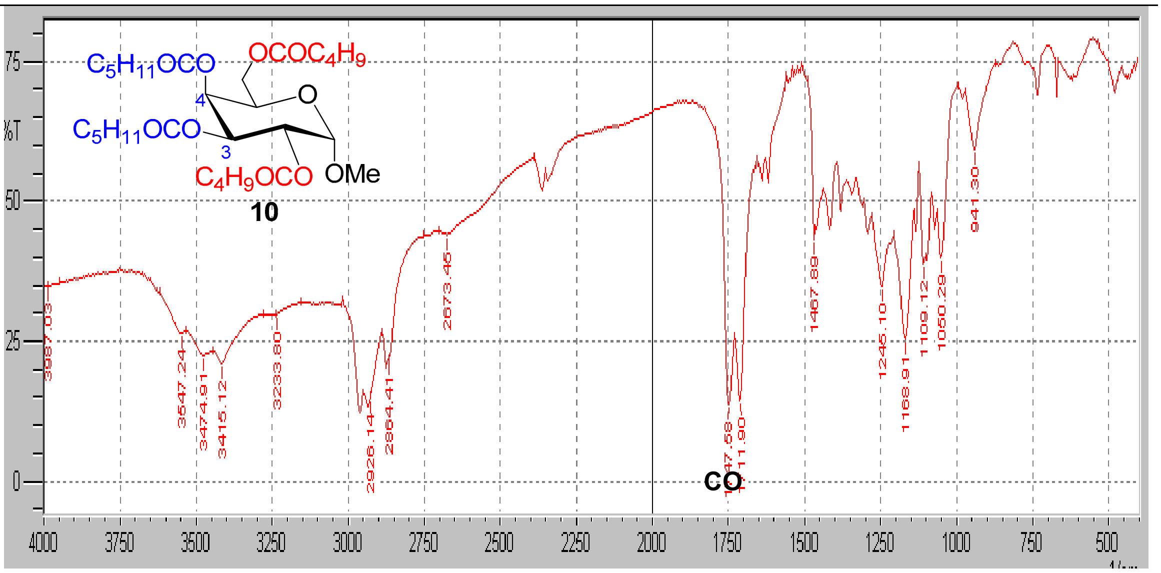

Figure S36. FT-IR (KBr) spectrum of compound **10**.

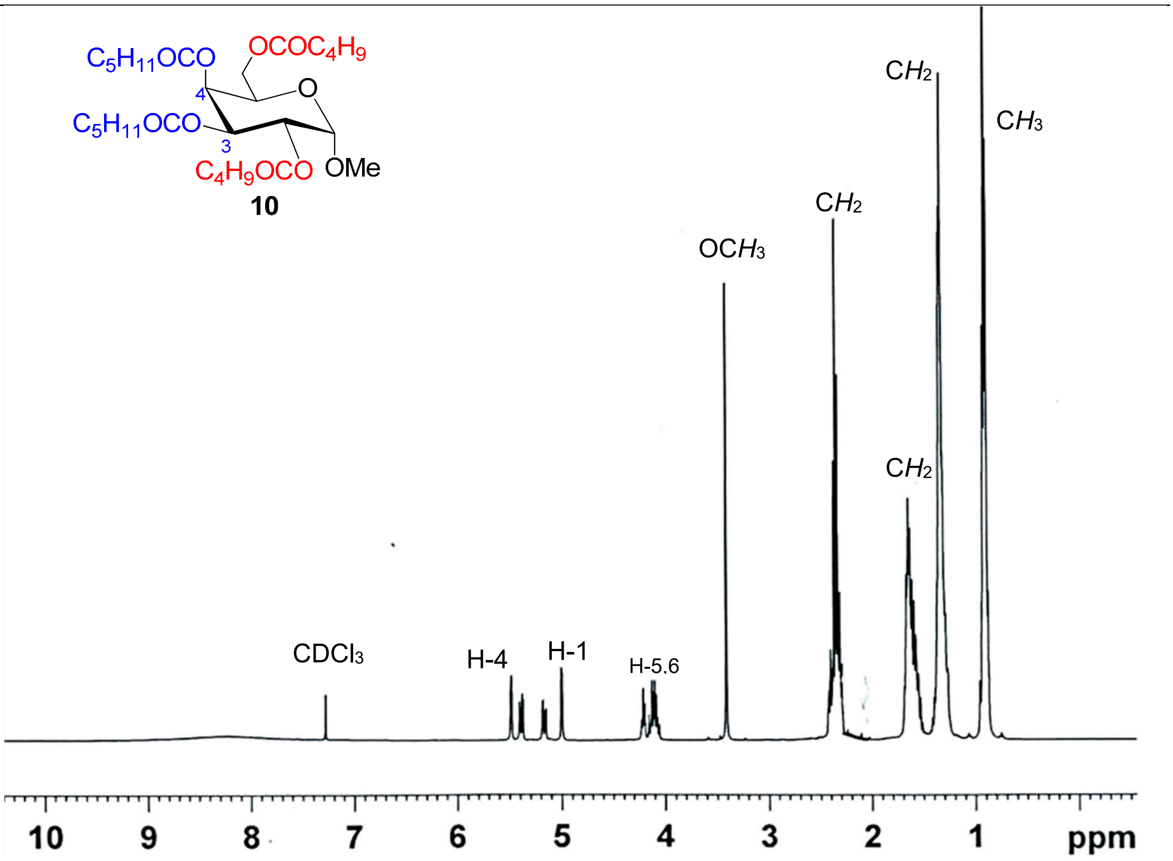

Figure S37. ^{1}H NMR (400 MHz, $CDCl_3$) spectrum of compound **10.**

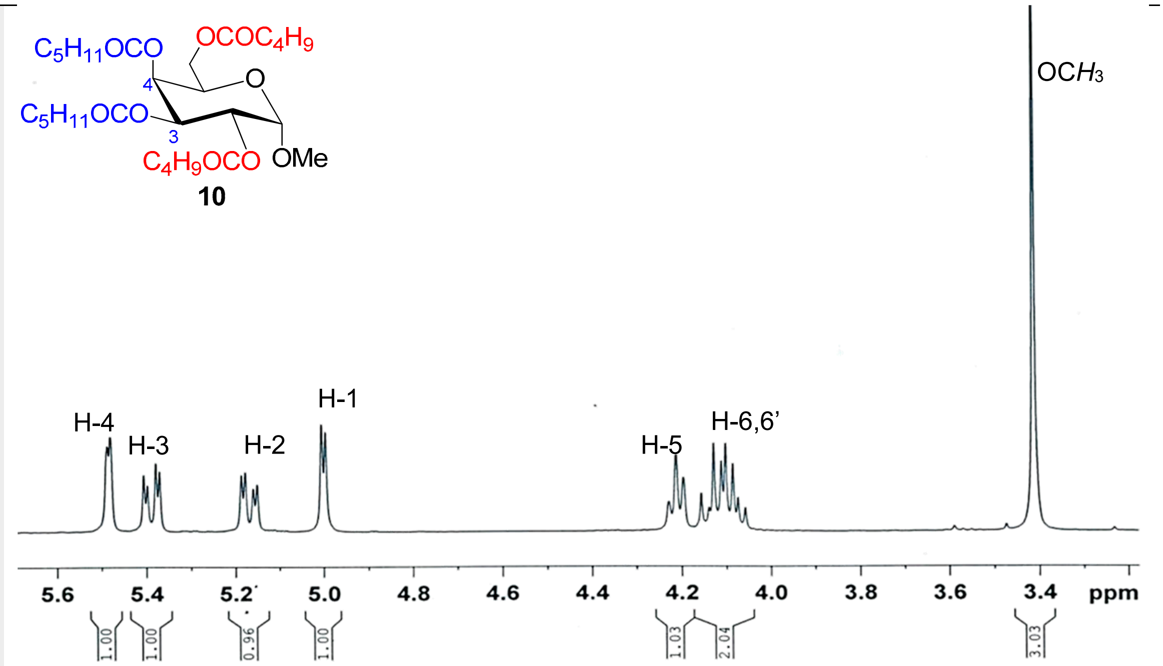

Figure S38. ^{1}H NMR (400 MHz, $CDCl_3$) spectrum of compound **10** (expansion).

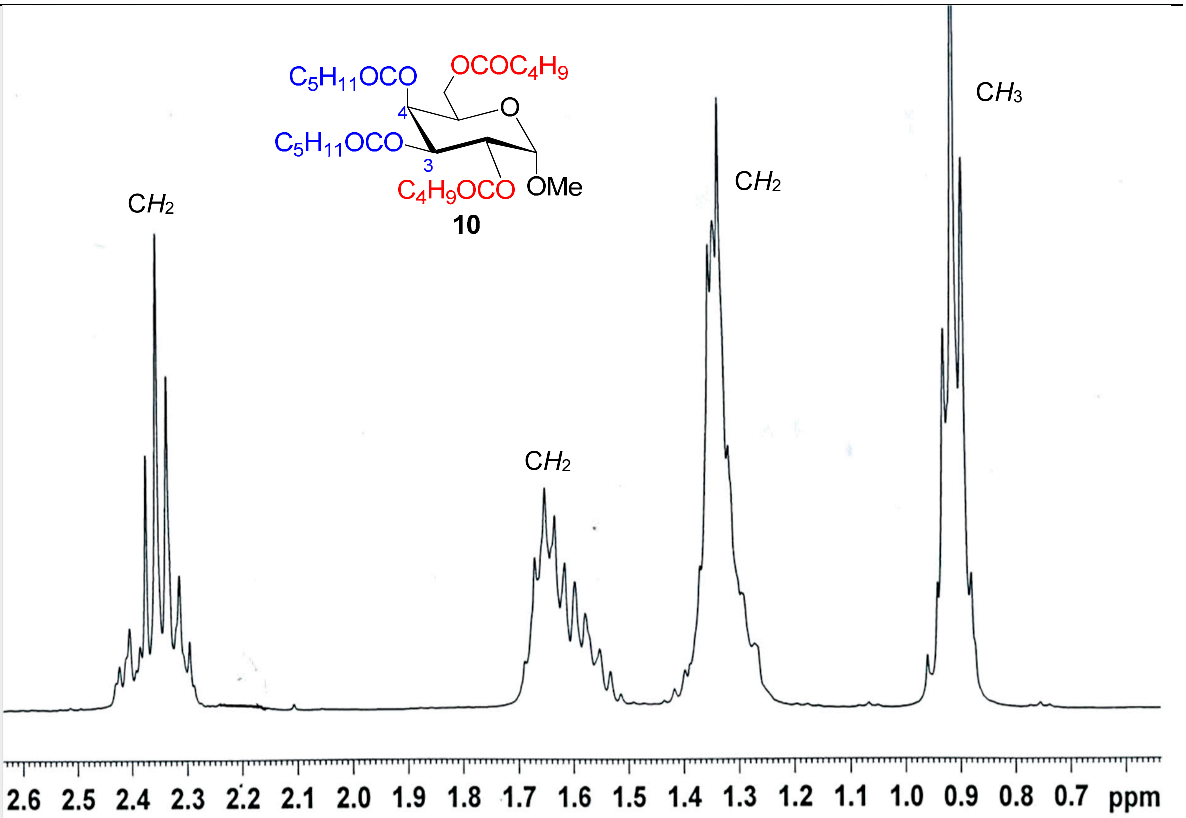

Figure S39. ^{1}H NMR (400 MHz, $CDCl_3$) spectrum of compound **10** (expansion).

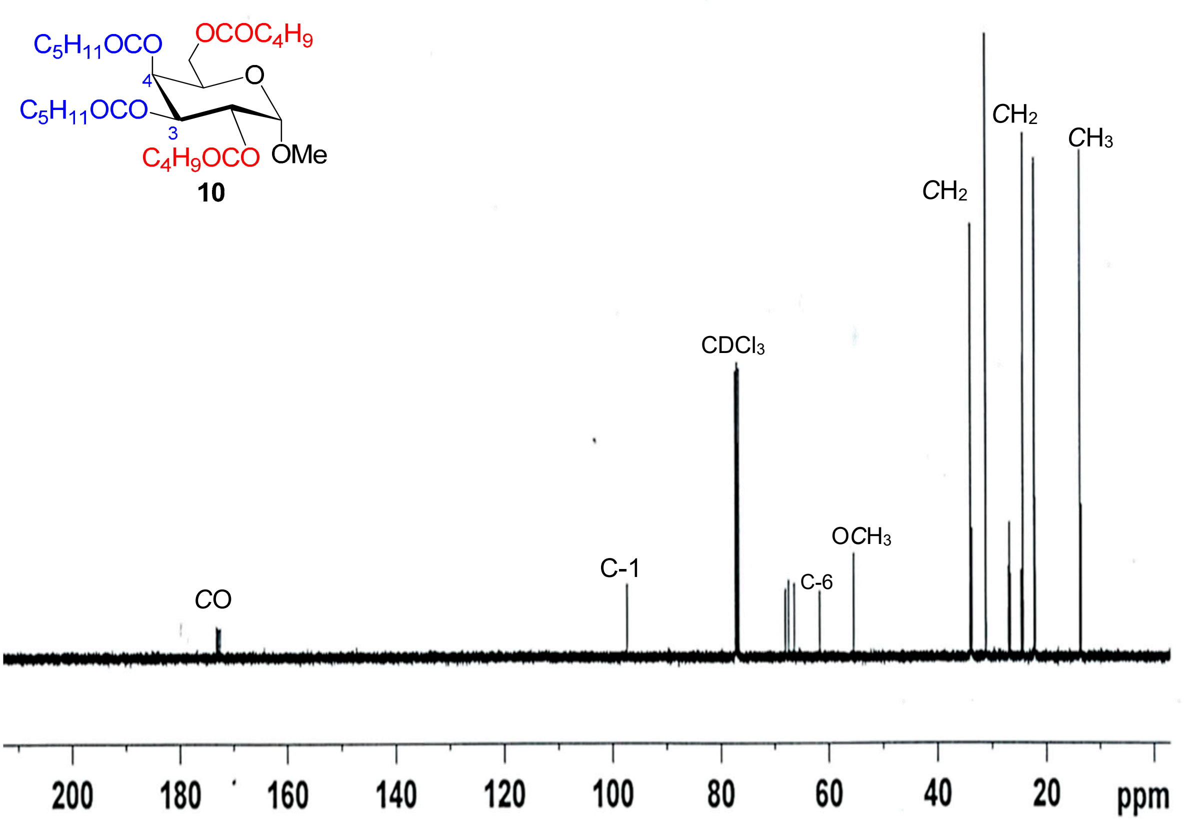

Figure S40. ^{13}C NMR (100 MHz, $CDCl_3$) spectrum of compound **10**.

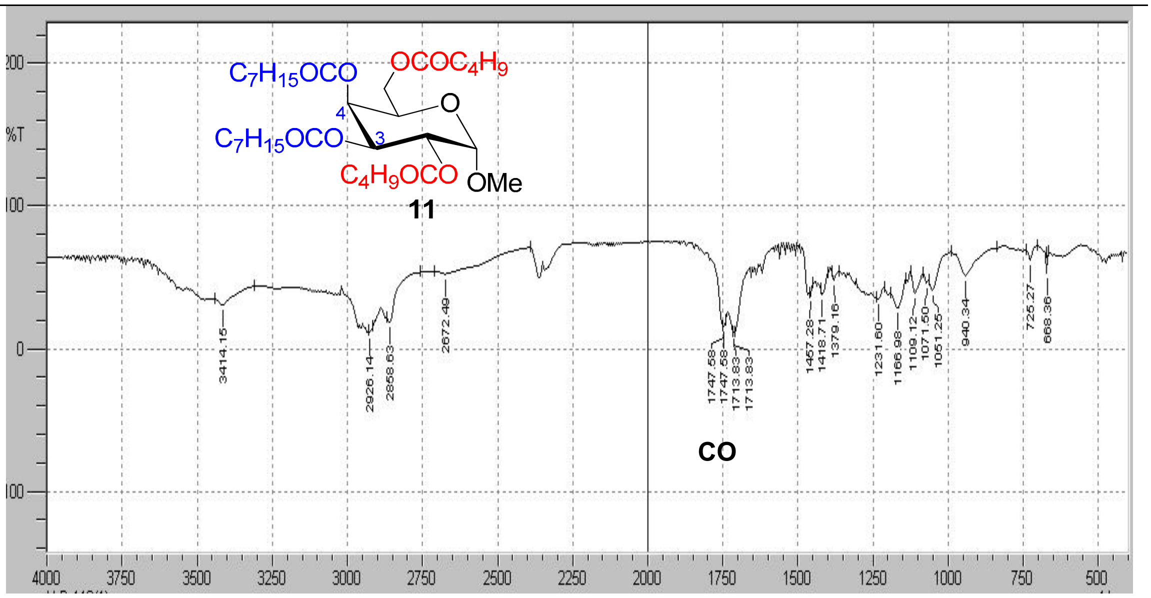

Figure S41. FT-IR (KBr) spectrum of compound **11**.

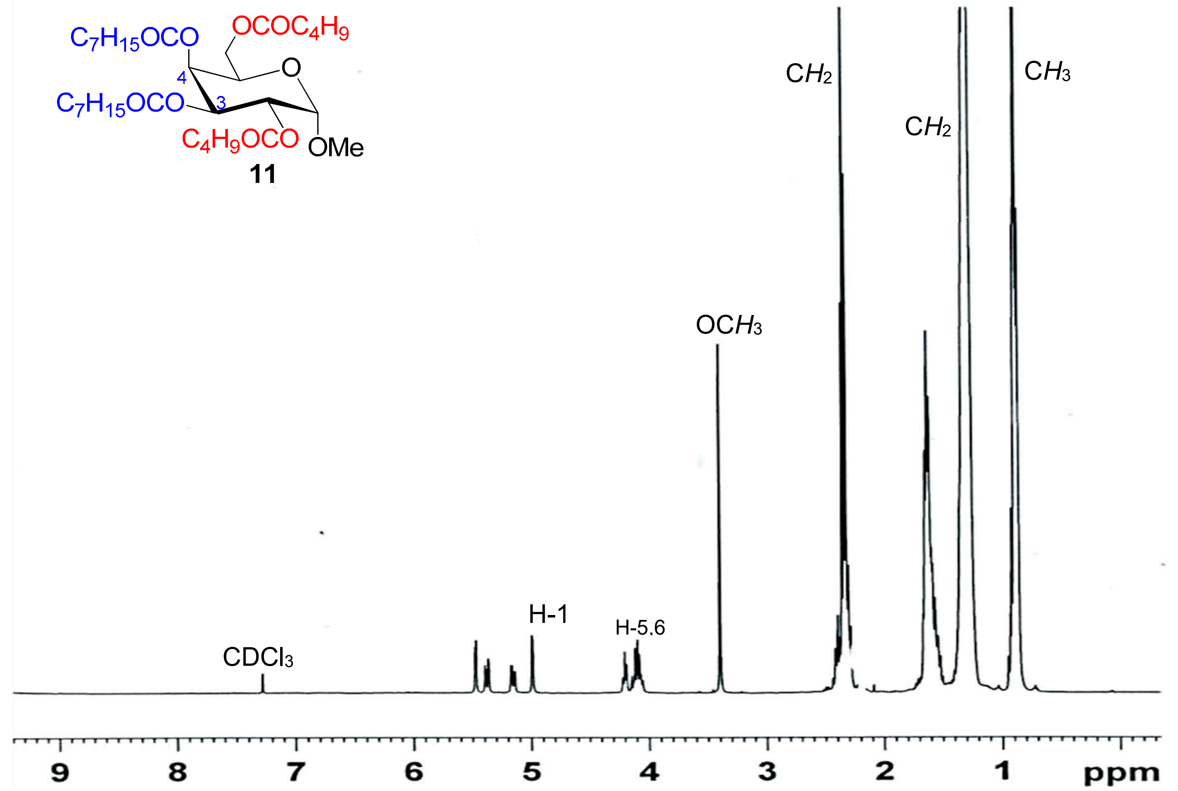

Figure S42. ^{1}H NMR (400 MHz, $CDCl_3$) spectrum of compound **11.**

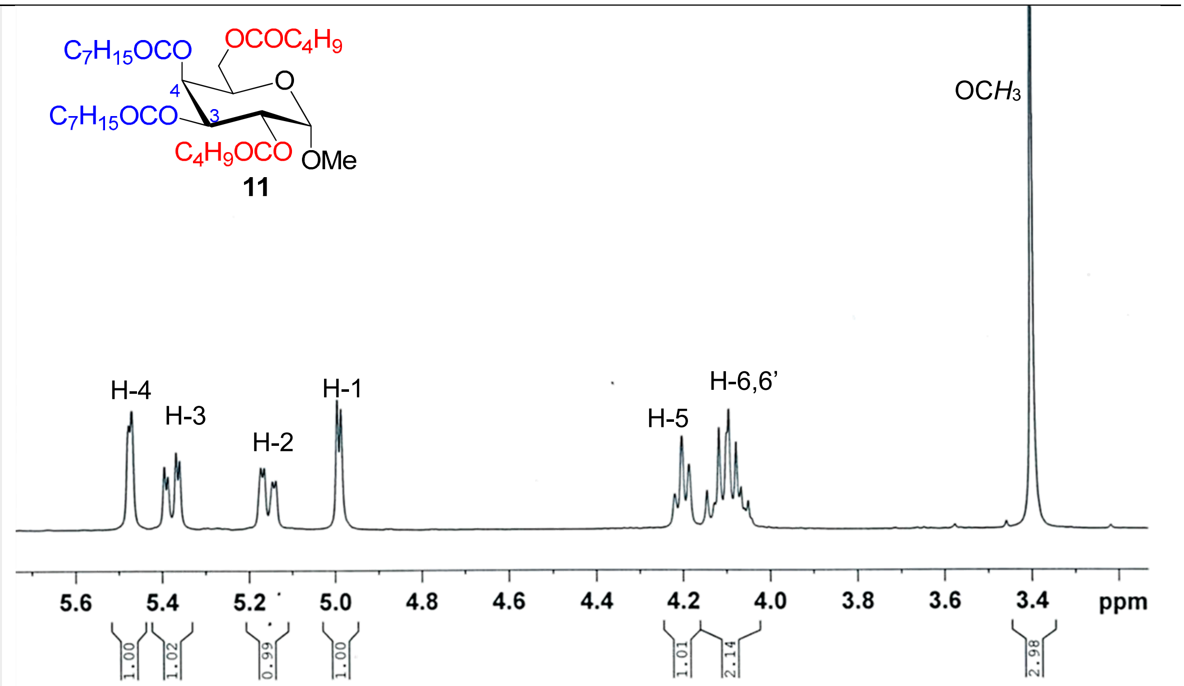

Figure S43. ^{1}H NMR (400 MHz, $CDCl_3$) spectrum of compound **11** (expansion).

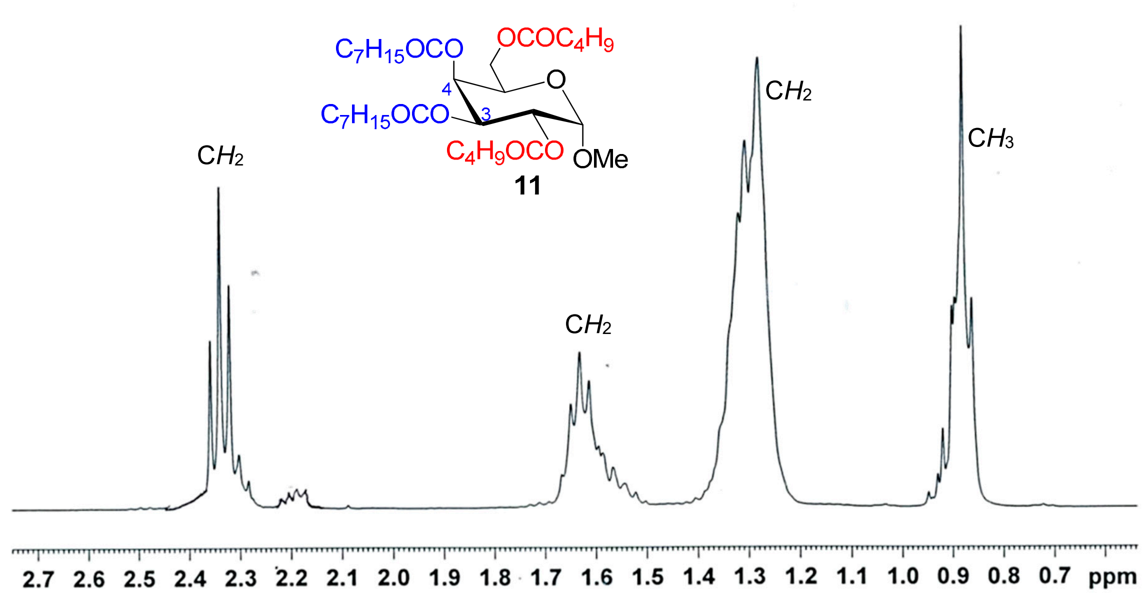

Figure S44. ^{1}H NMR (400 MHz, $CDCl_3$) spectrum of compound **11** (expansion).

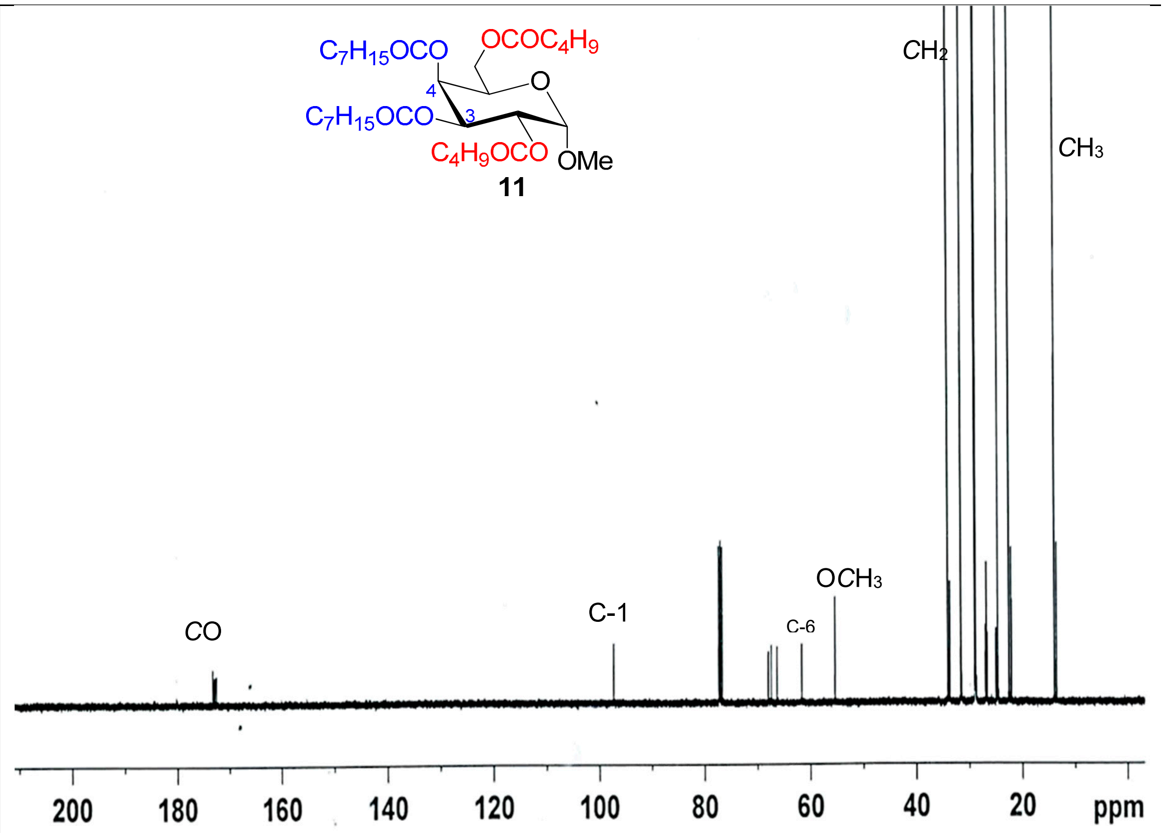

Figure S45. ^{13}C NMR (100 MHz, $CDCl_3$) spectrum of compound **11**.

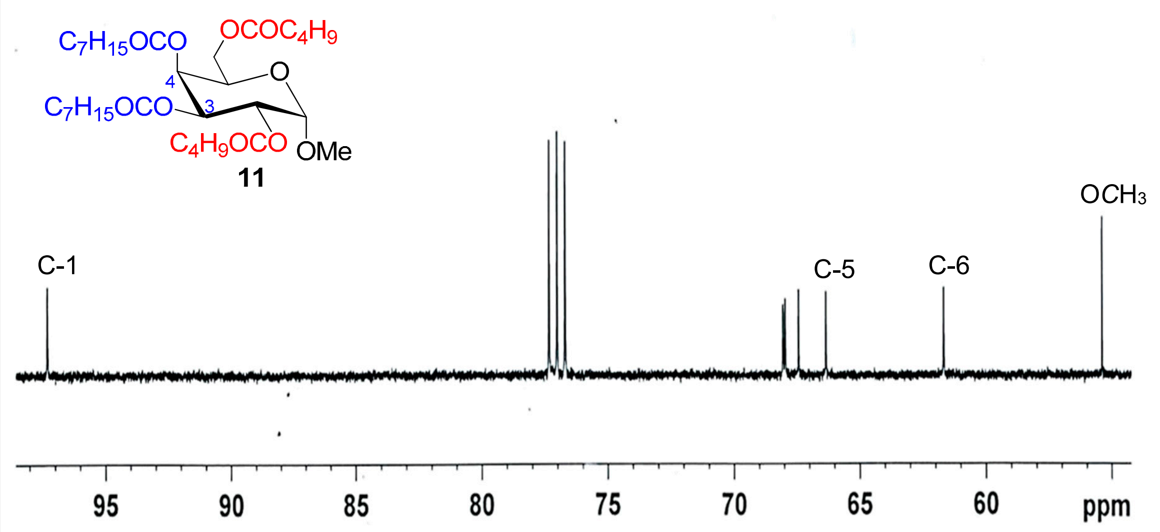

Figure S46. ^{13}C NMR (100 MHz, $CDCl_3$) spectrum of compound **11** (expansion).

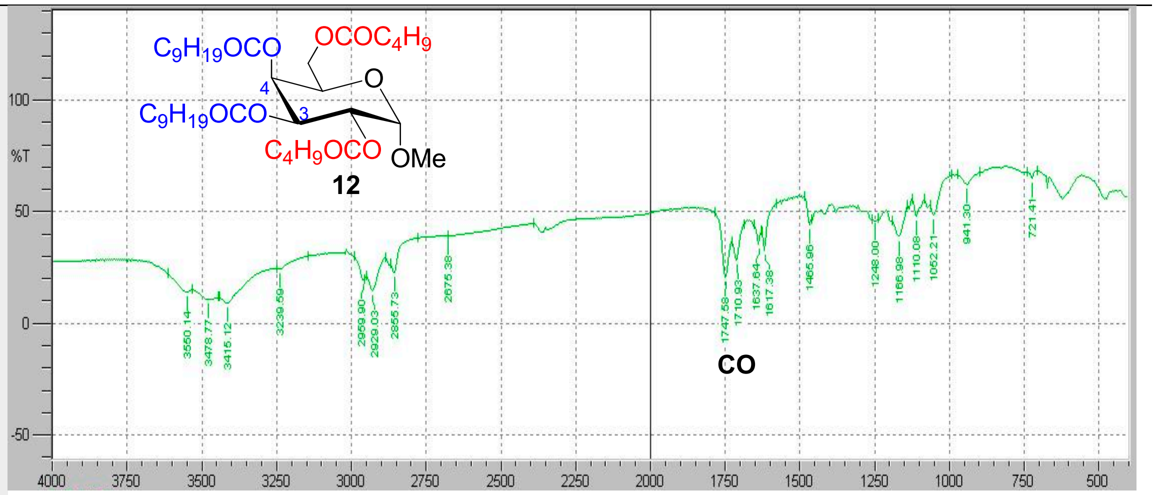

Figure S47. FT-IR (KBr) spectrum of compound **12**.

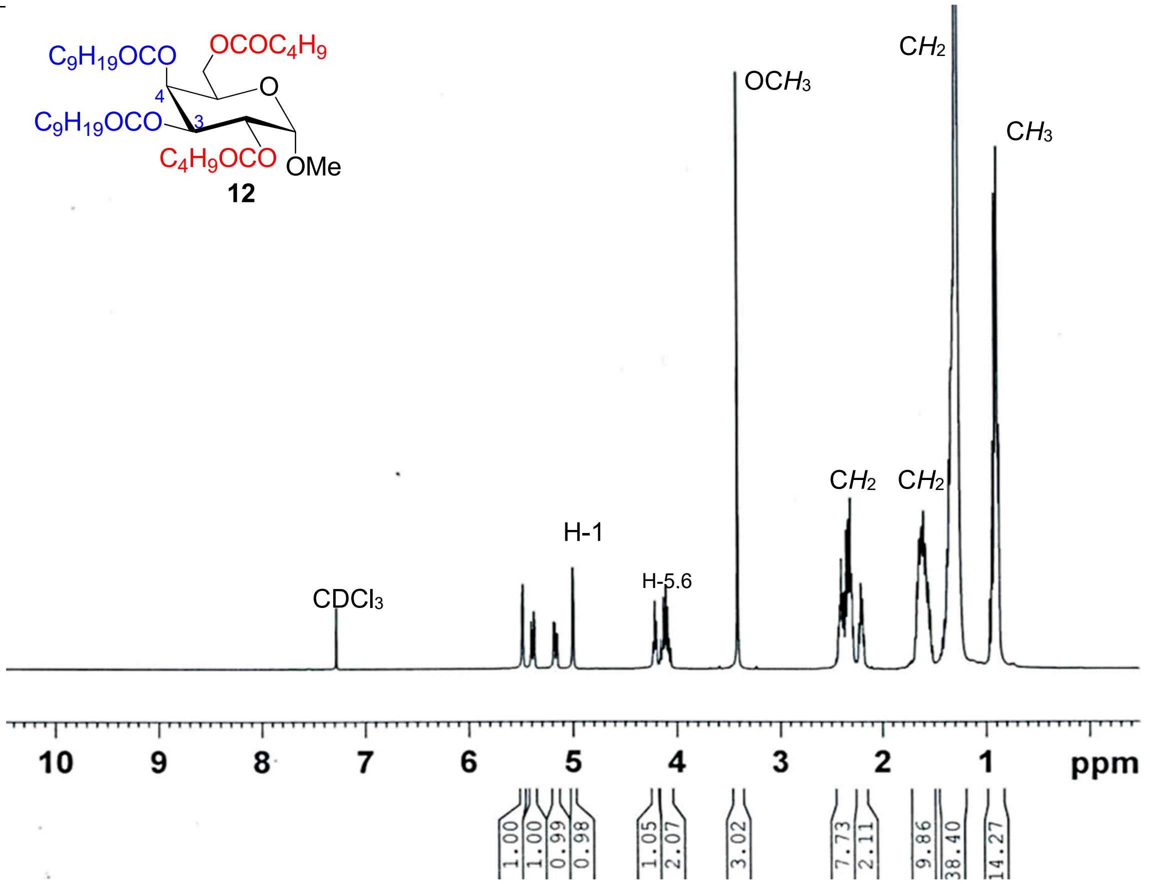

Figure S48. ^{1}H NMR (400 MHz, $CDCl_3$) spectrum of compound **12.**

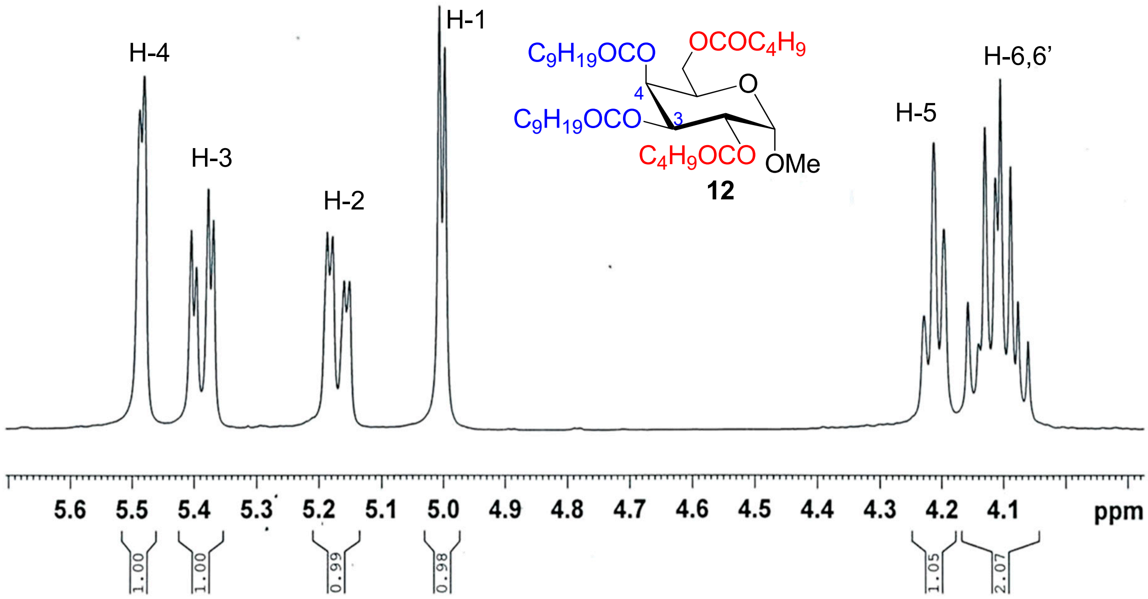

Figure S49. ^{1}H NMR (400 MHz, $CDCl_3$) spectrum of compound **12** (expansion).

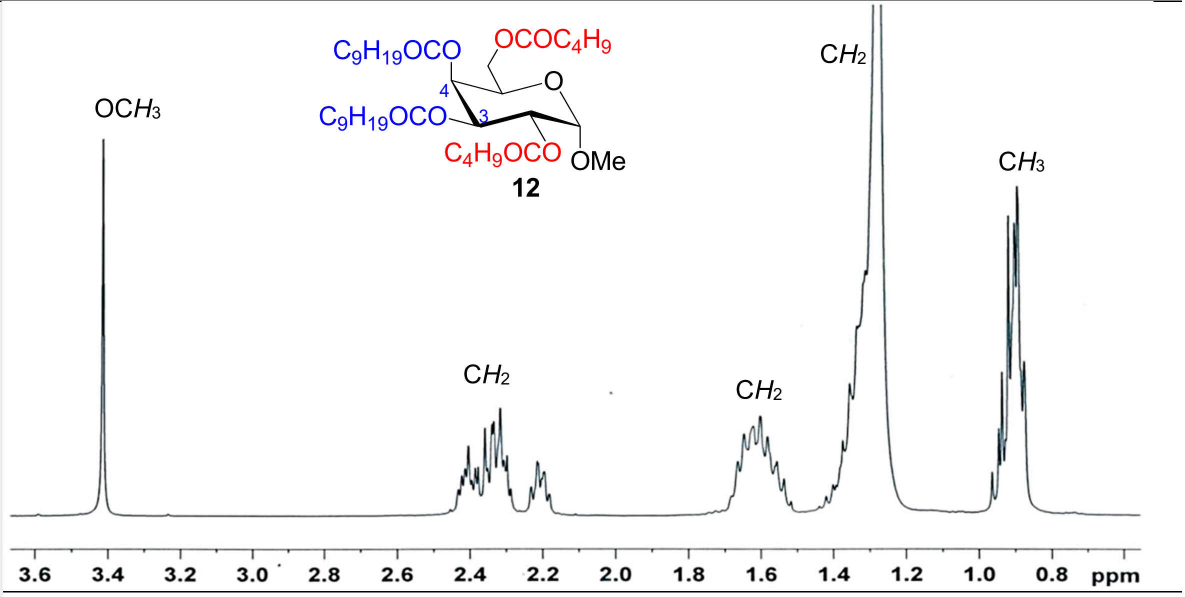

Figure S50. ^{1}H NMR (400 MHz, $CDCl_3$) spectrum of compound **12** (expansion).

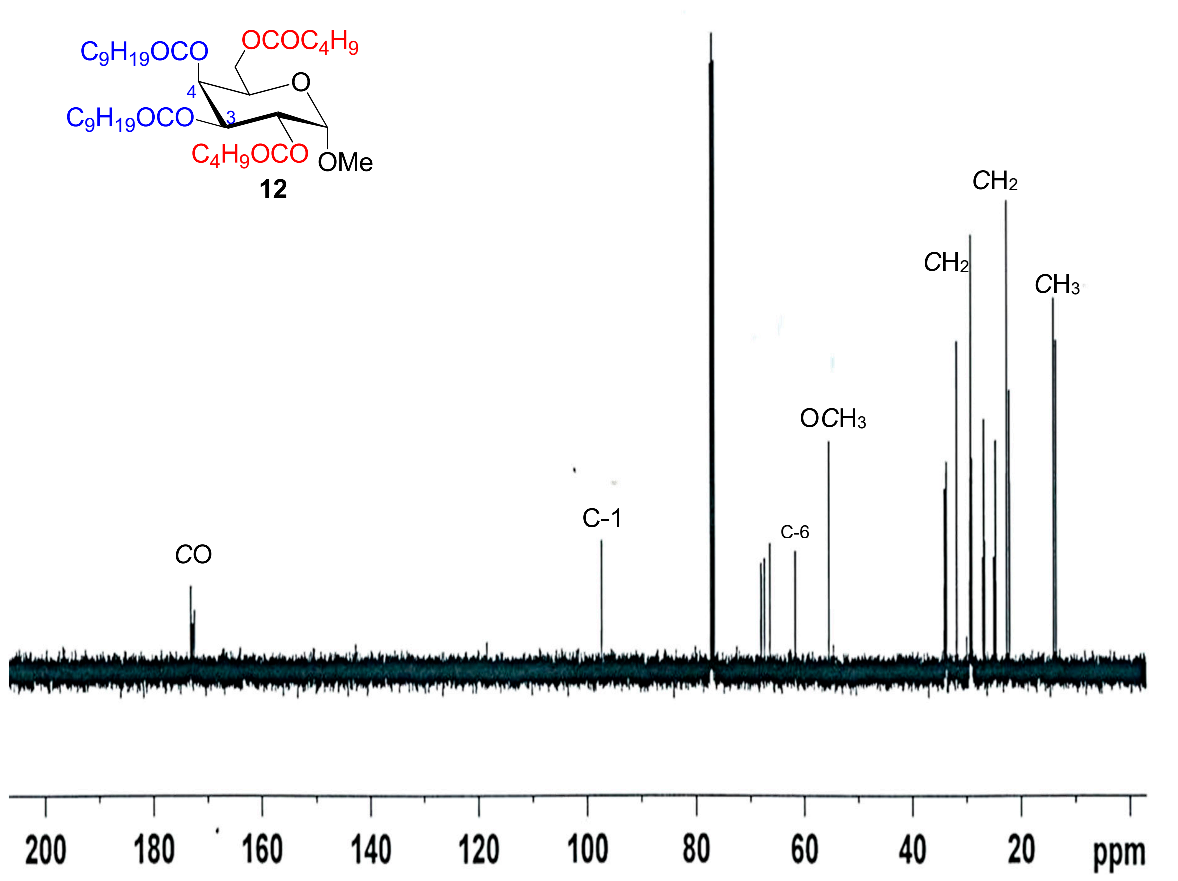

Figure S51. ^{13}C NMR (100 MHz, $CDCl_3$) spectrum of compound **12**.

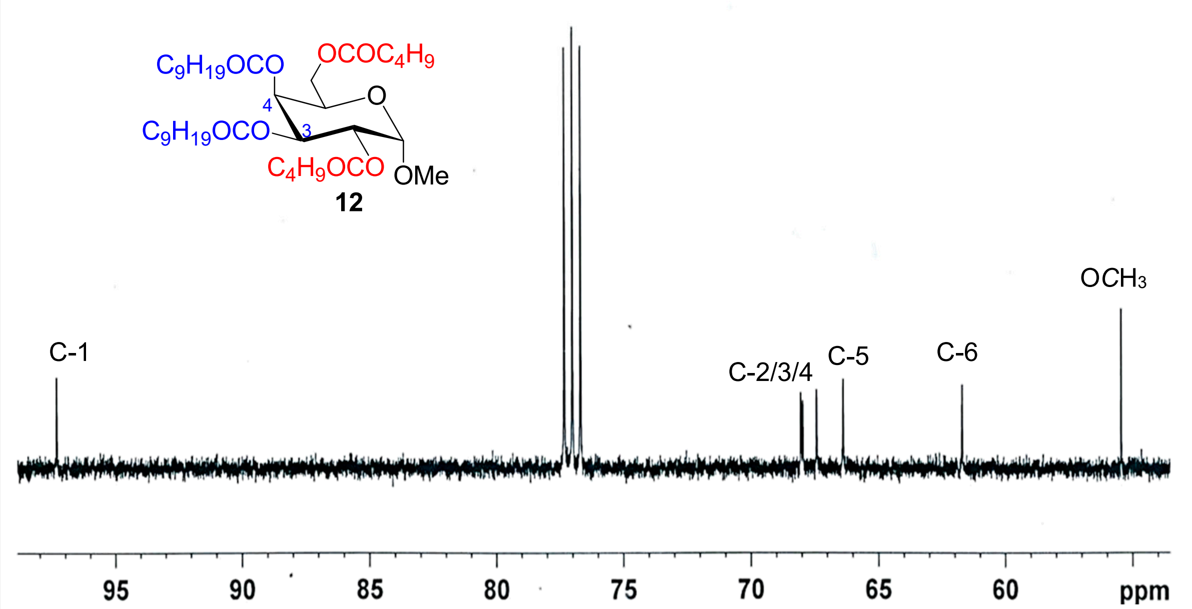

Figure S52. ^{13}C NMR (100 MHz, $CDCl_3$) spectrum of compound **12** (expansion).

Effects of test chemicals against bacteria

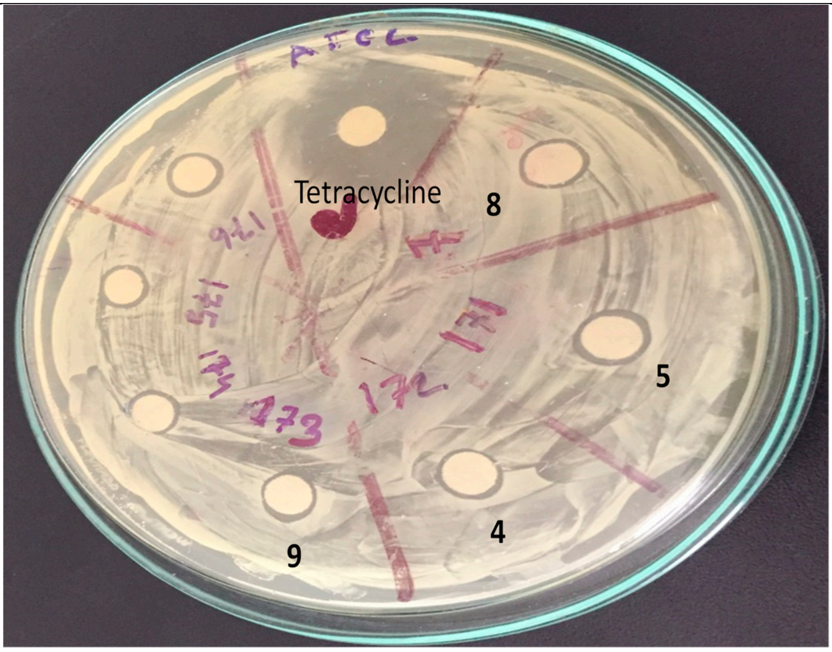

Figure S53. Inhibition against *Staphylococcus aureus* by 4,5, 8, 9 and tetracycline.

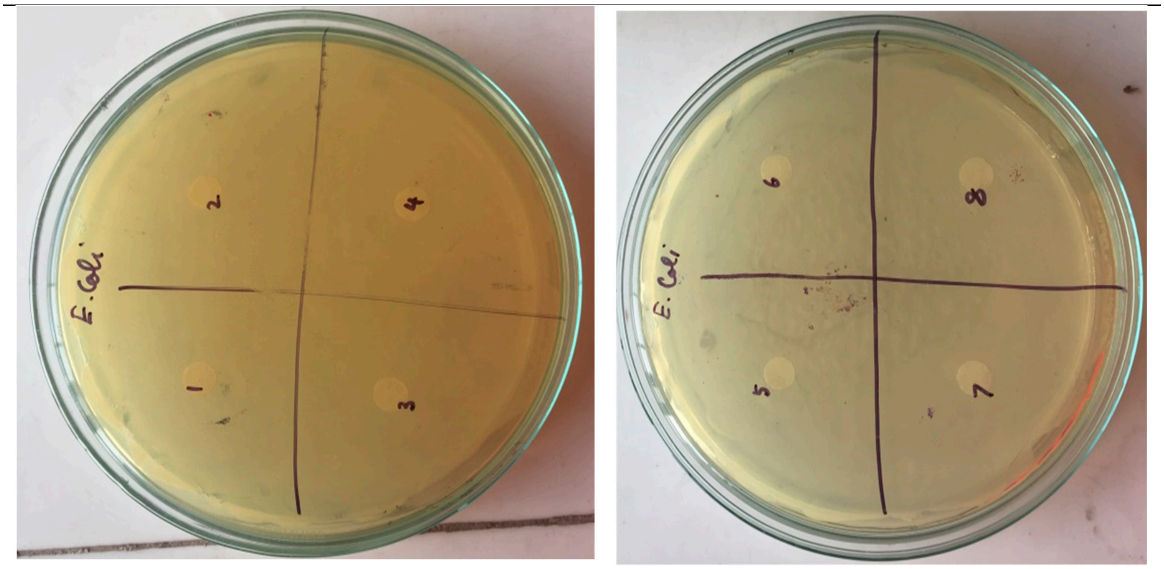

Figure S54. Galactopyranosides **3** and **4-12** showed against *E. Coli***.**

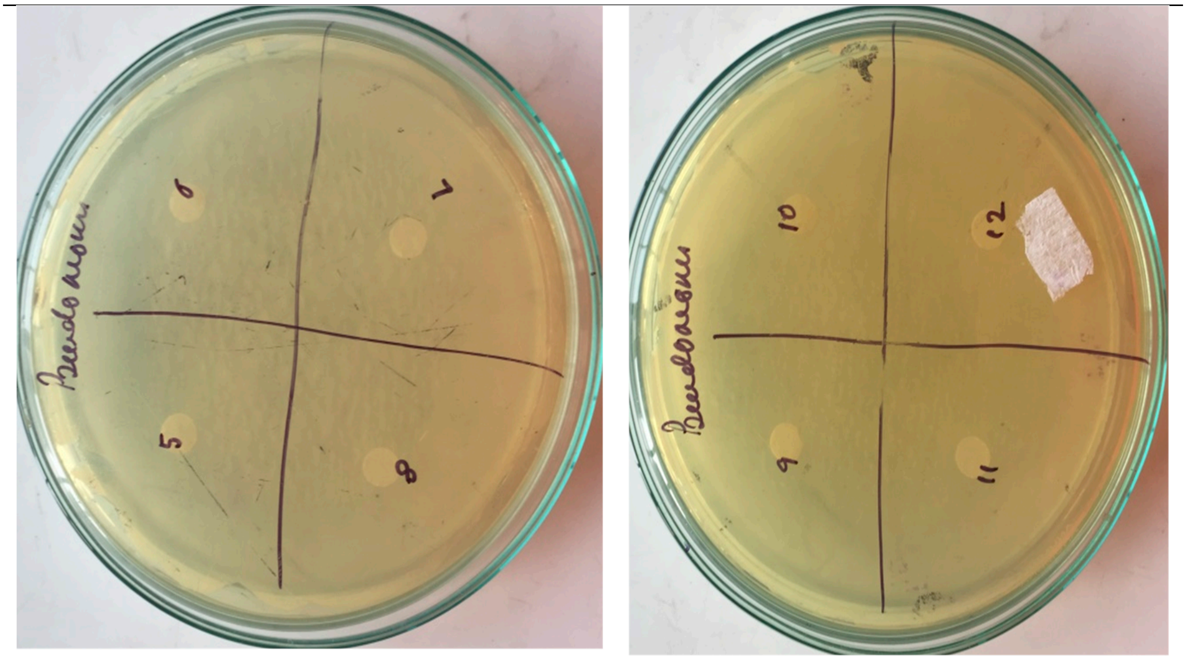

Figure S55. Galactopyranosides **3** and **4-12** showed against *P. aeruginosa***.**

Effects of test chemicals against fungi

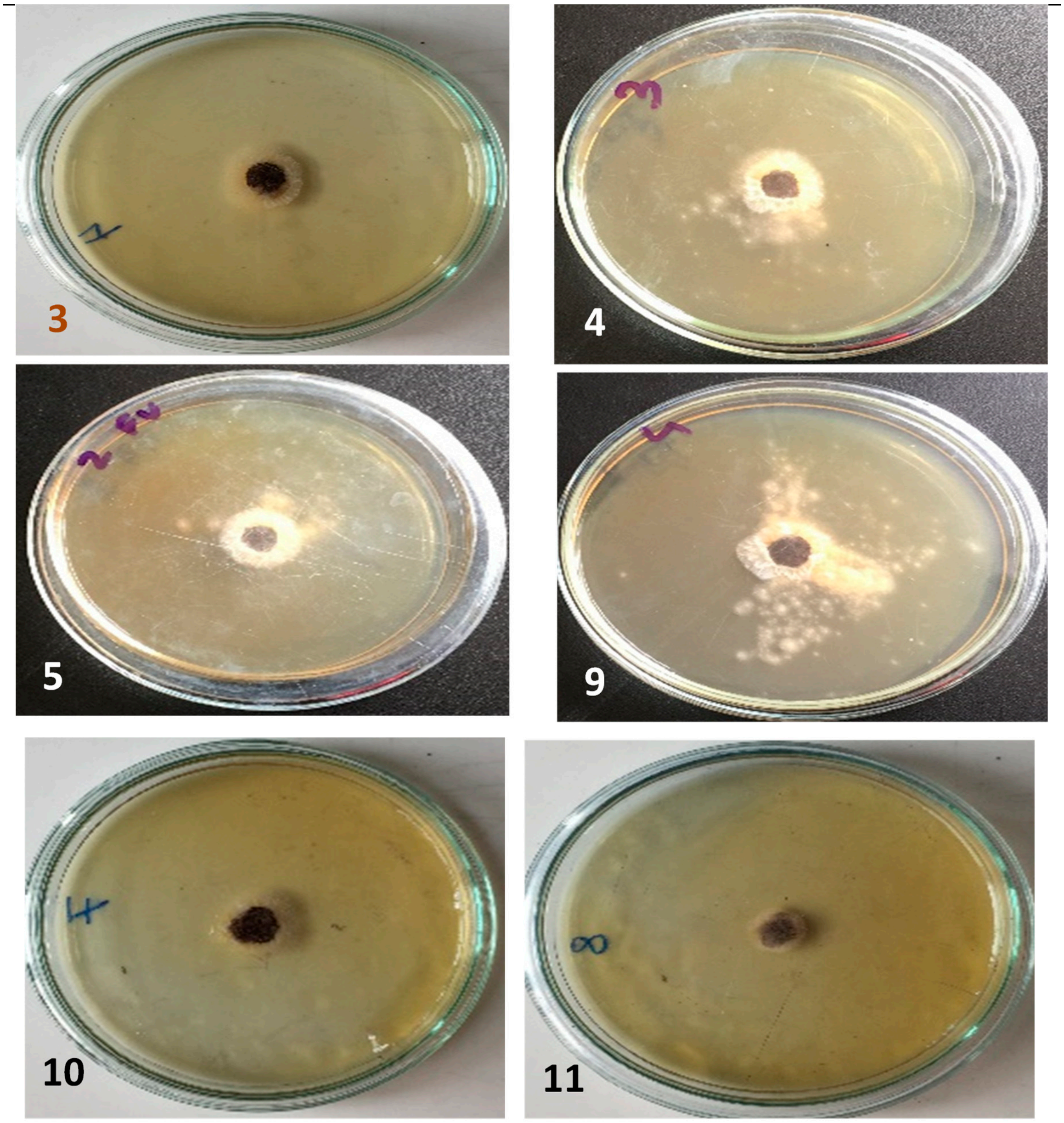

Figure S56. Zone of inhibition (%) against *Aspergillus fumigatus*

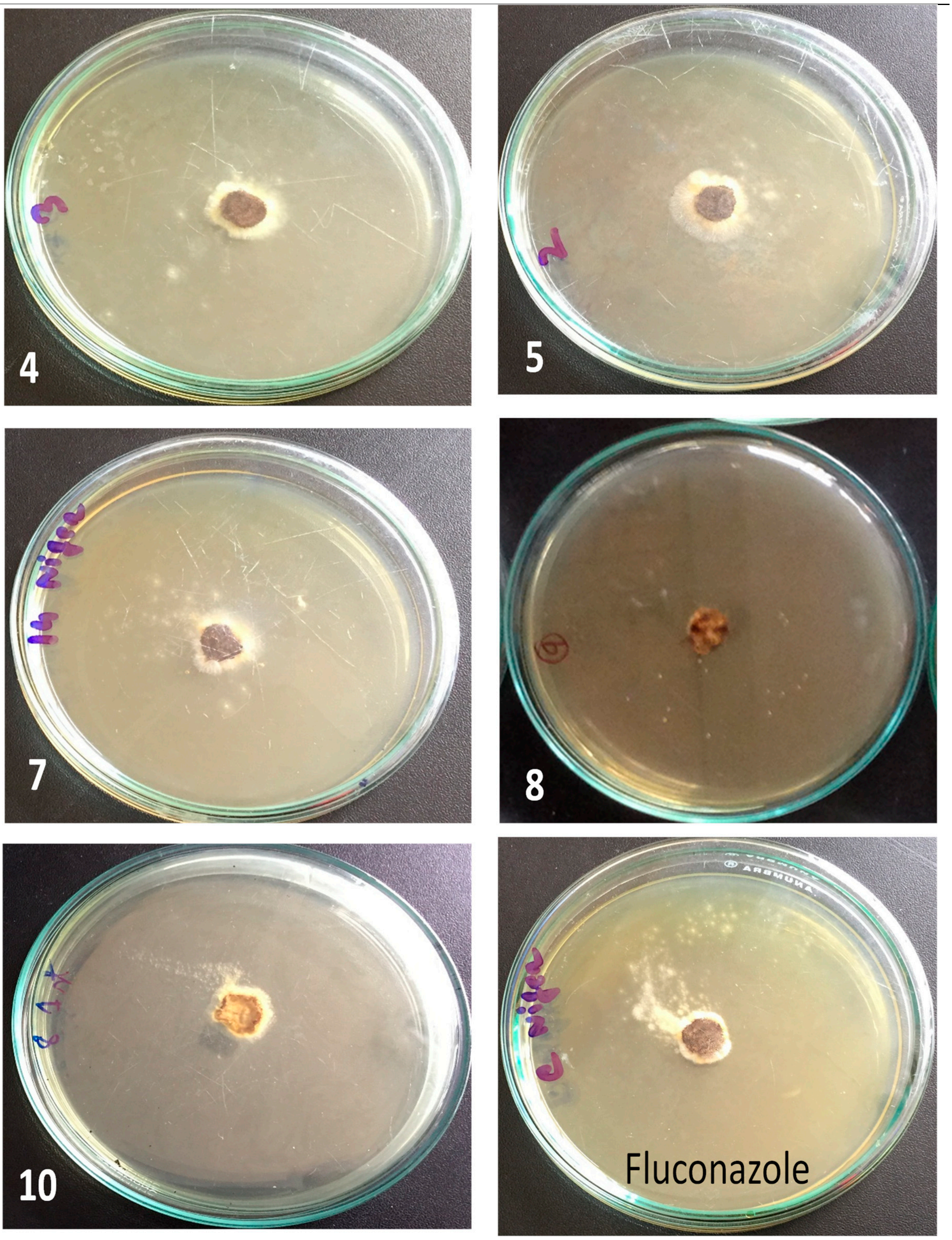

Figure S57. Zone of inhibition (%) against *Aspergillus niger*

Table S1: MM-GBSA Binding affinity calculation of sterol 14α-demethylase and SEs complexes.

Compounds	ΔG_{Bind}[a]	$\Delta G_{Bind\ Coulomb}$[b]	$\Delta G_{Bind\ Covalent}$[c]	$\Delta G_{Bind\ Hbond}$[d]	$\Delta G_{Bind\ Lipo}$[e]	$\Delta G_{Bind\ SolvGB}$[f]	$\Delta G_{BindvdW}$[g]
**VNI**	-83.93	110.51	3.6	-0.6	-40.76	-94.45	-58.9
**8**	-82.83	-3.12	10.49	-0.04	-45.94	32.61	-76.82
**13**	-80.63	-17.05	4.28	-2.13	-26.1	33.38	-73.02
**7**	-78	-25.46	11.6	-1.88	-28.48	33.17	-66.96
**14**	-72.48	-9.01	9.85	-2.85	-26.41	25.87	-69.86
**10**	-68.35	-12	12.62	-0.34	-32.45	25.2	-61.37
**12**	-65.25	-5.55	11.04	-0.24	-43.21	36.87	-64.17
**9**	-61.59	-37.72	9.41	-3.45	-14.56	33.41	-48.67
**11**	-59.24	0.48	16.15	0	-34.58	27.64	-68.92
**6**	-50.35	-36.05	6.96	-3.3	-7.27	30.67	-41.34
**Fluconazole**	-46.58	-7.48	3.08	-0.64	-16.26	15.41	-37.36
**Tetracycline**	-45.91	-20.71	3.31	-0.96	-18.61	40.37	-48.34
**5**	-39.63	-9.72	18.81	-3	-20.45	19.27	-44.53
**Tetracycline**	-35.89	-78.07	1.6	-0.86	-14.36	103.45	-46.63
**4**	-34.01	-14.49	5.39	-0.97	-16.48	22.7	-30.15
**Tetracycline**	-29.91	-132.73	3.23	-0.95	-14.79	163.05	-46.76
**3**	-25.33	-19.16	1.8	-1.81	-10.06	20.57	-16.67

[a]MM-GBSA free energy (kcal/mol) of binding.
[b]Contribution to the MMGBSA affinity of binding (kcal/mol) from the Coulomb energy.
[c]Contribution to the MMGBSA affinity of binding (kcal/mol) from covalent binding.
[d]Contribution to the MMGBSA affinity of binding (kcal/mol) from hydrogen bonding.
[e]Contribution to the MMGBSA affinity of binding (kcal/mol) from lipophilic binding.
[f]Contribution to the MMGBSA affinity of binding (kcal/mol) from the generalized Born electrostatic solvation energy.
[g]Contribution to the MMGBSA affinity of binding (kcal/mol) from the van der Waals energy.

Table S2: MM-GBSA Binding affinity calculation of SARS-CoV-2 main protease (6LU7) and SEs complexes.

Compounds	ΔG_{Bind}[a]	$\Delta G_{Bind\ Coulomb}$[b]	$\Delta G_{Bind\ Covalent}$[c]	$\Delta G_{Bind\ Hbond}$[d]	$\Delta G_{Bind\ Lipo}$[e]	$\Delta G_{Bind\ SolvGB}$[f]	$\Delta G_{BindvdW}$[g]
**N3**	-62.54	-18.08	6.45	-1.5	-16.9	37.45	-67.02
**12**	-61.9	-19.01	9.08	-1.52	-19.93	31.87	-62.4
**5**	-53.21	-15.74	5.18	-1.99	-16.13	12.3	-36.83
**7**	-50.62	-12.69	4.82	-1.09	-17.67	29.7	-53.69
**8**	-50.12	-9.63	18.96	-1.3	-23.7	33.12	-67.58
**10**	-49.72	-13.78	9.63	-1.82	-17.82	32.26	-58.19
**4**	-48.6	-15.19	2.87	-2.3	-13.44	13.02	-33.56
**9**	-48.23	-19.87	9.54	-1.79	-12.46	23.32	-46.97
**11**	-47.96	-7.75	16.24	-1.53	-20.14	31.16	-65.94
**Tetracycline**	-38.65	-20.09	4.23	-2.35	-9.53	17.62	-27.6
**6**	-35.93	-13.14	7.45	-1.26	-11.13	25.81	-43.67

**14**	-34.87	-6.83	4.23	-1.26	-10.64	31.6	-49.09
**13**	-33.57	-16.22	13.56	-1.14	-20.72	33.83	-39.37
**3**	-31.68	-25.21	4.33	-2.59	-6.52	15.9	-17.59
**Fluconazole**	-29.63	-2.98	5.97	-0.83	-8.85	13.82	-34.29

[a]MM-GBSA free energy (kcal/mol) of binding.
[b]Contribution to the MMGBSA affinity of binding (kcal/mol) from the Coulomb energy.
[c]Contribution to the MMGBSA affinity of binding (kcal/mol) from covalent binding.
[d]Contribution to the MMGBSA affinity of binding (kcal/mol) from hydrogen bonding.
[e]Contribution to the MMGBSA affinity of binding (kcal/mol) from lipophilic binding.
[f]Contribution to the MMGBSA affinity of binding (kcal/mol) from the generalized Born electrostatic solvation energy.
[g]Contribution to the MMGBSA affinity of binding (kcal/mol) from the van der Waals energy.
